# Integrating Genomic and Climate Data to Design Representative Seed Production Areas: A Pragmatic Workflow for Climate‐Adjusted Provenancing

**DOI:** 10.1002/ece3.72658

**Published:** 2026-01-15

**Authors:** Richard J. Dimon, Jason Bragg, Patrick Fahey, Marlien van der Merwe, Peter D. Wilson, Robert Henry, Maurizio Rossetto

**Affiliations:** ^1^ Research Centre for Ecosystem Resilience Royal Botanic Garden Sydney Sydney New South Wales Australia; ^2^ Queensland Alliance of Agriculture and Food Innovation University of Queensland St Lucia Queensland Australia; ^3^ Queensland Herbarium and Biodiversity Science, Department of the Environment, Tourism, Science and Innovation Brisbane Botanic Gardens Toowong Queensland Australia; ^4^ School of Natural Sciences Macquarie University Macquarie Park New South Wales Australia

**Keywords:** climate‐adjusted provenancing, ex situ conservation, genetic diversity, sampling optimisation, seed production areas, seed sourcing

## Abstract

Establishing genetically diverse ex situ collections, particularly seed production areas (SPAs), is essential not only for safeguarding biodiversity but also for generating high‐quality and high‐quantity germplasm material. However, practical tools for sourcing genetically representative material remain limited, especially for widespread, common species. Here, we present a flexible, data‐driven workflow that integrates genomic data, future climate projections and real‐world constraints to guide the design of representative SPAs. Using the widespread rainforest tree *Neolitsea dealbata* as a case study, we identified genetic neighbourhoods (GNs) across its range and used a climate‐matching tool to pinpoint an *external GN* with a future climate analogous to a target restoration area (the Big Scrub). We evaluated how common allelic diversity is captured under three practitioner‐defined decisions: (1) whether to minimise individuals or sites sampled, (2) whether to apply sampling constraints and (3) whether to sample randomly or optimally. To support the third decision, we developed a novel optimisation method that identifies combinations of individuals or sites using a down‐projected site frequency spectrum (*psfs*), aiming to maximise allele representation in the final collection. These decisions were then implemented across three provenancing strategies: local, predictive and climate‐adjusted. Our results show that multiple sampling approaches can capture over 90% of common alleles (a predefined threshold) for the *local GN*, even under various logistical and practical constraints. The same is feasible when including future climate‐matched sources from an *external GN*, which nearly doubled allelic representation of the species in the SPA. This workflow is adaptable to practical limitations, such as site inaccessibility or reliance on existing collections. By balancing genetic resolution with practitioner flexibility, our approach supports scalable, evidence‐based design of ex situ collections, such as SPAs, to maximise genetic representation under environmental change.

## Graphical abstract

In this study, we present a flexible, data‐driven workflow that integrates genomic data, future climate projections and real‐world constraints to guide the design of genetically representative seed production areas (SPAs). We show how common allele representation within a target genetic neighbourhood can be applied across various sampling strategies involving local, predictive and climate‐adjusted provenancing.

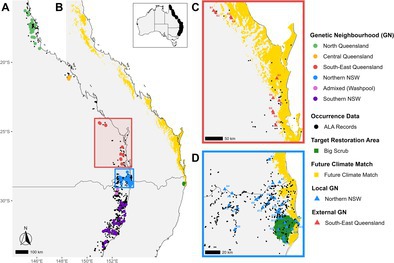

## 1 Introduction

Ecological restoration practices are increasingly guided by genetic evidence to inform sourcing from both local and climate‐resilient provenances (Broadhurst et al. 2023; Vitt et al. 2022). Establishing genetically representative populations, whether through ex situ collections or direct restoration, helps preserve evolutionary processes and ecological functions (Hoban et al. 2013), reduces inbreeding (Frankham 2015) and improves adaptive capacity and climate resilience (Harrison et al. 2021; Sgrò et al. 2011). Conversely, neglecting genetic diversity when sourcing material can lead to reduced fitness, lower resilience to fragmentation and environmental stress, and greater vulnerability to invasive species and pathogens (Broadhurst et al. 2023; Havens et al. 2015).

### 1.1 Genetically Informed Sampling Strategies

Genetic tools and guidelines for in situ germplasm sourcing help address key practical questions, such as where to collect material and how many individuals are needed to represent meaningful genetic diversity. Sourcing regions are often delineated by areas of gene flow and zones of genetic turnover, referred to as *genetic neighbourhoods* (Fahey et al. 2025; Rossetto et al. 2019), *eco‐sourcing regions* (Heenan et al. 2024) or *seed zones* (Heenan et al. 2024). Following Fahey et al. (2025), the term *genetic neighbourhood* (GN) is used here.

Sampling from multiple sites within a GN can increase genetic diversity while maintaining a balance between inbreeding and outbreeding depression. Examining population‐level metrics such as mating systems, kinship, inbreeding, differentiation and gene flow can further refine sampling strategies (Fahey et al. 2025; McKay et al. 2005). Once patterns of genetic structure and gene flow are understood, sourcing strategies can be tailored to specific goals: maximising local diversity, enhancing climate resilience (Prober et al. 2015) or combining both (Harrison et al. 2021). Accordingly, these provenancing scenarios are commonly defined as *local*, *predictive* or *climate‐adjusted* (see Table S1 for further examples).

Although restoration genomics is a developing field, several national initiatives now incorporate genetically informed provenancing, including the National Seed Strategy (Oldfield 2019) and Florabank Guidelines (Harrison et al. 2021), underpinned by web‐based tools such as the *Seedlot Selection Tool* and *Climate‐Smart Restoration Tool* (St. Clair et al. 2022) and the *Restore and Renew* webtool (Rossetto et al. 2019). These frameworks guide sourcing to capture both local diversity and climate‐adaptive potential. As environmental conditions shift, relying solely on local provenancing may be insufficient for long‐term resilience (Radchuk et al. 2019). Modern restoration strategies therefore aim to retain local genotypes while incorporating diversity predicted to perform well under future climates (Bragg et al. 2022; Jordan et al. 2024).

While genetic tools have advanced regional‐scale provenancing, approaches for selecting specific individuals or sites within these regions for ex situ collections remain limited. For threatened species, optimisation approaches have been developed to maximise representation of genetic diversity through targeted individual selection (Bragg et al. 2020). However, these methods are rarely applied to widespread, non‐threatened species commonly used in restoration. Moreover, approaches that integrate genetic optimisation with climate‐adjusted provenancing (balancing local genetic representation with inclusion of external, climate‐matched genotypes) are still largely unexplored for widespread taxa (Breed et al. 2019).

### 1.2 Establishing Genetically Representative Seed Production Areas

Seed Production Areas (SPAs) offer a scalable approach to improving both the supply and quality of plant material for ecological restoration and conservation. As ex situ collections, SPAs provide a sustainable seed source that reduces pressure on wild populations (Zinnen et al. 2021), forming a vital link between conservation genetics and on‐ground restoration.

Genetically representative SPAs aim to capture not only the genetic diversity of target restoration areas but also the adaptive and evolutionary potential of species in changing environments. However, every management decision, from the choice of founding material to seed production and collection methods, can influence the genetic composition of the resulting stock (Basey et al. 2015). Incorporating population genetics to guide SPA design offers a robust, repeatable framework to build collections that reflect species' evolutionary histories while maintaining representative diversity across target regions.

Quantifying allelic diversity provides a powerful way to assess how well different SPA sampling strategies capture evolutionary potential. The widespread acceptance of representing broad genetic diversity as a proxy for adaptive capacity (Batista et al. 2016; Lind and Lotterhos 2025) enables practitioners to both quantify and optimise ex situ collections using all available genetic markers for long‐term restoration and conservation outcomes. Previous studies have explored how sampling design influences allele representation, comparing random versus optimised sampling (van der Merwe et al. 2021), maternal lineage coverage (Schumacher et al. 2024), population structure (Hoban and Schlarbaum 2014), sample size per site (Marshall and Brown 1975) and strategies to buffer against allele loss through individual mortality (Hoban 2019). Collectively, these studies emphasise the need for frameworks that balance genetic representativeness with practical feasibility.

Despite these advances, designing representative SPAs remains challenging, especially across broad geographic scales where cost, logistics and genetic goals must be balanced. As SPAs are increasingly adopted, the development of standardised, data‐driven and flexible workflows that integrate genetic tools with stakeholder priorities will be essential to achieve both scalability and long‐term genetic representativeness.

### 1.3 Logistical Considerations for Practitioners

Translating genetic principles into clear, actionable guidance for restoration practitioners remains challenging, even as large‐scale, multi‐species initiatives continue to expand globally (Rossetto et al. 2019; St. Clair et al. 2022; Wood et al. 2024). Although the scientific foundations for genetic representativeness are well established, practical implementation often faces barriers such as difficult field access, propagation constraints and administrative burdens that can lead to the deprioritisation of genetic goals during on‐ground activities (Török et al. 2024).

SPAs offer a promising framework for reconciling these challenges by linking local decision‐making with broader genetic objectives (Zinnen et al. 2021). However, differences in practitioner expertise, site accessibility and temporal availability to source in situ material for establishing an SPA underscore the need for flexible sourcing strategies that maintain genetic representativeness while remaining operationally feasible. Pre‐identified plants or sites may also become inaccessible through mortality, lost tags or new collection restrictions. In some cases, suitable germplasm, such as reproductively mature individuals, may not be available at target sites (FAO 2025). The sheer scale of multi‐site sampling can further constrain collection efforts, particularly when establishing SPAs simultaneously across multiple species with differing biological or phenological traits. Once material enters nurseries, additional challenges arise, including limited propagation space, consistent accession labelling and the management of multi‐species record systems, all of which can strain capacity and resources.

Addressing these constraints requires sampling strategies that balance practicality with genetic breadth. Approaches that enable collection from fewer sites or individuals, while still capturing representative variation, can greatly improve feasibility. Flexible designs that allow substitution of equivalent sites or germplasm types also offer pragmatic solutions for scaling restoration across species and regions.

### 1.4 Study Objectives

This study aims to provide a robust, replicable workflow for sourcing genetically representative germplasm to establish ex situ collections such as SPAs. Building on the framework of Bragg et al. (2020) for threatened species translocations, where genetically optimised populations are established by selecting specific individuals, we extend these concepts to widespread species, for which recollecting genotyped individuals may not necessarily be feasible.

Our approach focuses on capturing spatial patterns of genetic diversity, rather than targeting known genotypes, to establish genetically diverse and representative collections. To achieve this, the workflow comprises three key steps: (1) identification of local and external genetic neighbourhoods (GNs) relevant to a target restoration area; (2) integration of practitioner‐led decision‐making to capture representative diversity while accounting for practical constraints such as geographic scale, accessibility and nursery capacity and (3) application of provenancing scenarios that represent local diversity, external diversity (e.g., for climate resilience) or a combination of both.

To guide development of this workflow, we address the following questions: (1) Can *local* and *external GN*s be identified for a target species relative to a defined restoration area? (2) What sampling combinations best capture representative genetic diversity while accommodating logistical constraints? and (3) How can these combinations be applied across provenancing scenarios to balance local and external diversity?

We demonstrate the workflow through a case study involving a widespread rainforest tree species, where an SPA is required for downstream restoration projects within a defined target area. This application builds on previous frameworks (Fahey et al. 2025; Rossetto et al. 2019) to develop a scalable, data‐driven approach for designing genetically representative SPAs. The workflow incorporates new methods for regional climatic modelling and a novel optimisation tool that identifies site and individual combinations using a down‐projected site frequency spectrum (*psfs*).

A key assumption of this study is the flexibility of SPA size. Some SPAs may face fixed capacity limits, while others may want to support substantially larger collections. The focus here is to determine the minimum number of individuals and sites required to capture representative diversity within a defined GN.

## 2 Methods

### 2.1 Sampling, Data Generation and Filtering

**FIGURE 1:**
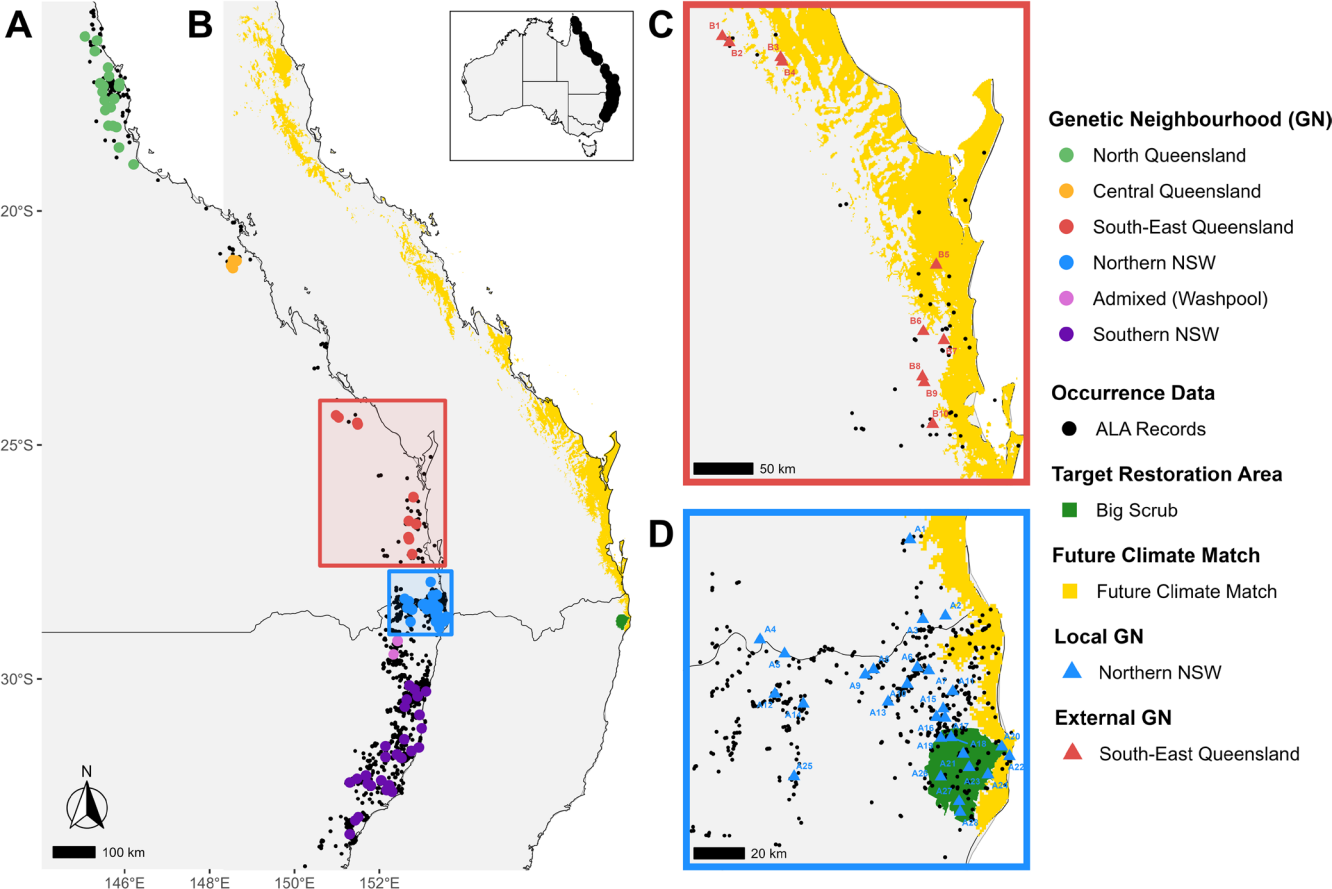
Series of maps to illustrate (A) The distribution of *Neolitsea dealbata* (black dots); occurrence data from the Atlas of Living Australia (ALA 2025) and the sampling sites for genetic analysis, with each estimated GN indicated in a different colour. (B) The areas (yellow) currently experiencing the climate that the Target Restoration Area (green; Big Scrub) is predicted to experience in 2070 based on modelling using the climate‐matching tool. (C) The external Genetic Neighbourhood (*external GN*) in closest proximity to the Local Genetic Neighbourhood (*local GN*) of the Target Restoration Area where the sites that overlap with the modelled future climate occur within the yellow areas. (D) The extent of the *local GN* of the Target Restoration Area (green area). Further analyses supporting the identification of GNs presented here are outlined in Figure 3 and Figures S5 and S6 in Appendix 2.

We applied our workflow to a population genomic dataset of *Neolitsea dealbata* (R.Br.) Merr., a common, broadly distributed small rainforest tree commonly used in ongoing restoration projects across eastern Australia (pers. comm., Big Scrub Rainforest Conservancy, 2025). Population‐level sampling, DNA sequencing and data filtering followed the standardised workflow of the Restore and Renew knowledge infrastructure project (Rossetto et al. 2019). Briefly, leaf material was collected from five to six individuals per site, ensuring a minimum spacing of 10 m between samples. Sampling sites were selected to represent the species' extant distribution, from the wet tropics of northern Queensland to the southern limit of its range on the south coast of New South Wales, Australia (Figure 1). Sites were defined as locations with at least five individuals and separated from other sites by a minimum of 3 km.

For DNA extraction, approximately 100 mg of leaf tissue per individual was sent to Diversity Arrays Technologies Pty Ltd. (Canberra) for DArTseq genotyping‐by‐sequencing data generation (Jaccoud et al. 2001; Kilian et al. 2012). The resulting single nucleotide polymorphism (SNP) dataset was processed using the dartR v2.9.7 package (Gruber et al. 2018) and an accompanying in‐house R package RRtools, available at https://github.com/jasongbragg/RRtools/, as part of the Restore and Renew analytical pipeline. All analyses were conducted in RStudio v2024.12.0 + 467 using R v4.3.1 (R Core Team 2021; RStudio Team 2020). SNP dataset filtering retained loci with reproducibility scores > 0.96 and < 20% missing data across samples. Loci containing more than one SNP were subsampled to retain a single SNP per locus. Samples missing over 50% of loci and sites with fewer than five remaining individuals were excluded.

**FIGURE 2:**
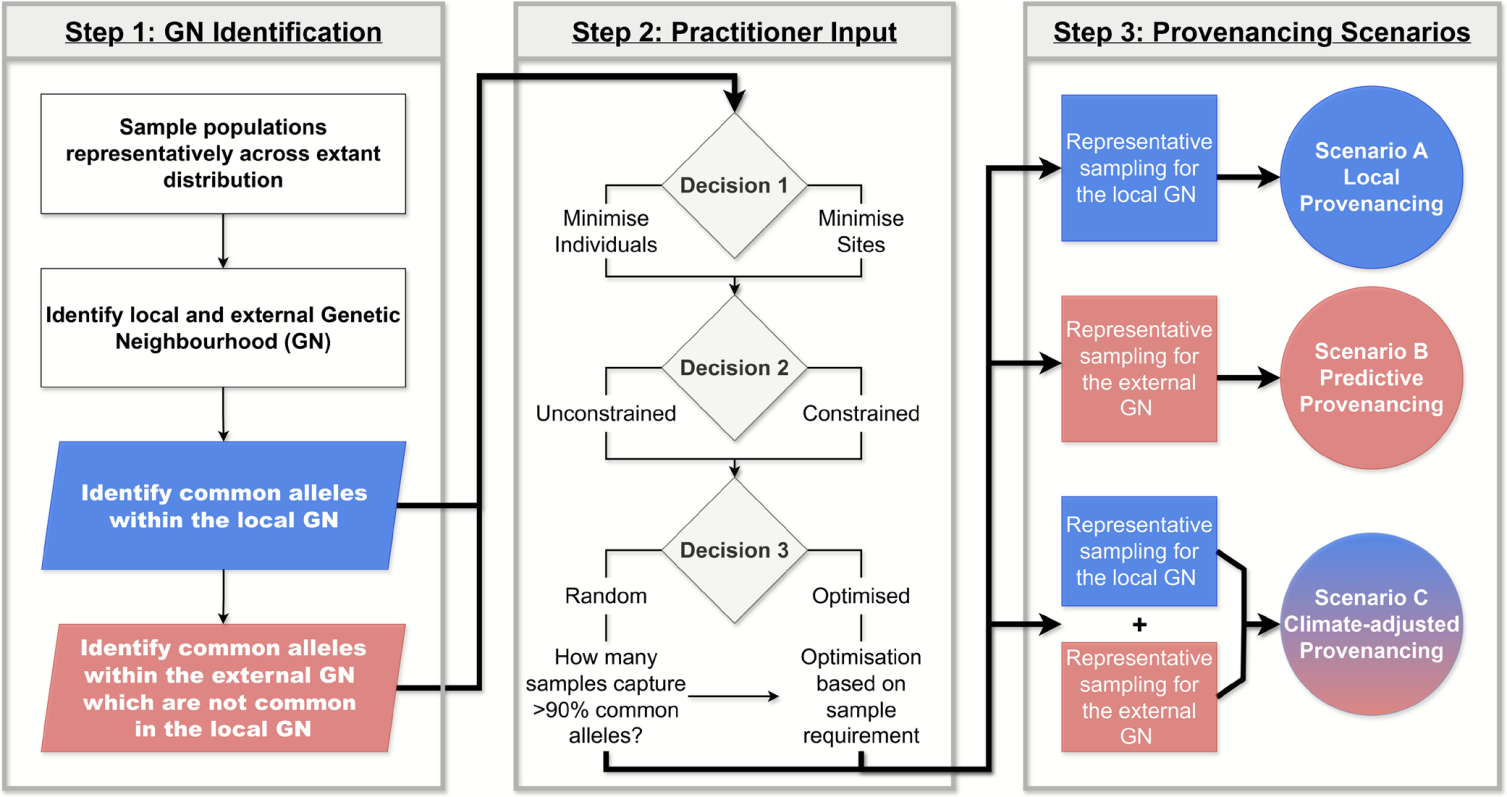
Workflow for establishing a genetically representative germplasm collection across three provenancing strategies, outlining the key decision‐making steps guided by practitioner input to accommodate logistical challenges and sampling constraints. Processes coloured blue involves targeting the identified local genetic neighbourhood (*local GN*), while red involves the identified *external GN*.

The below methods outline three major steps to achieve representative sampling: (1) GN Identification; (2) practitioner input and (3) provenancing scenarios. These steps are visualised in the workflow presented in Figure 2.

### 2.2 Step 1: Identification of Genetic Neighbourhoods

A series of preliminary analyses were conducted to assess population dynamics and identify GNs across the sampled distribution, following a similar framework outlined by Fahey et al. (2025). Highly similar samples within each site were first identified using pairwise kinship estimates based on the PLINK method, implemented via the *snpgdsIBDMoM* function in the SNPRelate v1.33.1 package (Zheng et al. 2012), with a minor allele frequency (MAF) threshold of ≥ 0.05. Individuals were considered genetically identical when their estimated kinship coefficient exceeded 0.354 (Manichaikul et al. 2010), with only a single representative from each identical pair retained to avoid redundancy. Sites with fewer than five individuals remaining after filtering were subsequently excluded from downstream analyses.

To assess whether genetic variation across the landscape followed a pattern of isolation‐by‐distance (IBD), pairwise *F*_ST_ values were calculated between all sites using SNPRelate and compared against geographic distances (km) via a Mantel test with 999 permutations, as implemented in the vegan v2.6–4 package (Oksanen et al. 2022). Multiple clustering approaches were then applied to the genotype matrix to identify GNs. Principal component analysis (PCA) was conducted using adegenet v2.1.9 (Jombart and Ahmed 2011), and network visualisation was generated using Splitstree v4.17.2 (Huson and Bryant 2006) via the NeighborNet method. Further validation of GN boundaries and detection of admixture regions were performed using the LEA v3.10.2 package (Frichot and François 2015), implementing the *sNMF* (sparse non‐negative matrix factorisation) algorithm. The optimal number of ancestral populations (K = 5) was selected based on post‐stabilisation of cross‐entropy values and visual inspection of each LEA analysis, following the approach of Rutherford et al. (2022).

Together, these complementary clustering methods provided a robust delineation of GNs for subsequent provenancing strategies. Sites where the highest average LEA ancestry proportion was < 60% were classified as admixed, representing zones of gene flow between divergent groups. To ensure accurate capture of common alleles within each target GN, admixture zones were excluded from GN boundaries and downstream analyses. While such admixed sites may still inform sampling strategies (particularly when they occur within a target restoration area), including them in this case risks obscuring the allele structure characteristic of the core ancestral groupings within each GN.

### 2.3 Identification and Allele Classification of the *Local* and *External GN*

For this study, the Big Scrub lowland subtropical rainforest (NSW, Australia; Figure 1) was designated as the target restoration area. The GN encompassing this region was defined as the *local GN*, and all sites within it were considered relevant for sampling under the local provenancing scenario. Alleles within the *local GN* were classified as either common or rare based on their frequency in the genotype dataset. Minor allele frequency (MAF); the frequency of the less common allele at each locus, was calculated from the genotype data (including missing loci) using the get_minor_allele_frequencies R function (see associated data repository). Alleles with MAF ≥ 5% were classified as *common*, while those < 5% were classified as *rare*.

To identify a suitable *external GN* representing populations with potential future climate adaptation, we developed a regional future climate‐matching analysis. This approach extends the *Restore and Renew* climate‐matching webtool (Rossetto et al. 2019), which identifies areas across Australia where projected 2070 climatic conditions match those of a specified location. Rather than matching a single coordinate, we applied this method across the entire Big Scrub region, using the full range of observed minimum‐maximum climate values (see Appendix 1 for methodological details). This regionalised approach identifies present‐day areas within mainland Australia that experience climatic conditions projected for the Big Scrub by 2070, thereby guiding regional sampling of potentially climate‐resilient material.

Future climate conditions for the Big Scrub were modelled under a moderate emissions scenario (RCP 4.5), focusing on mean annual temperature and rainfall. Localities currently matching these projected conditions were identified, and the geographically nearest GN encompassing any of these “future climate habitats” was selected as the *external GN*. To minimise risks of outbreeding depression while also increasing additional genetic diversity, we ensured the chosen GN also exhibited pairwise *F*_ST_ values < 0.5 relative to *local GN* sites. Ultimately, the GN located immediately north of the *local GN* was identified as meeting these criteria, comprising sites that match future climatic conditions projected for the Big Scrub (Figure 1) and maintaining *F*_ST_ < 0.5 between local and external sites (Figure S6 in Appendix 2). After filtering, the *local GN* comprised 163 individuals across 28 sites, and the *external GN* comprised 58 individuals across 10 sites, with 5–6 individuals per site (Table S2 in Appendix 3).

This study aimed to illustrate how different provenancing strategies can be applied to develop an SPA that maximises representation of (1) common alleles found within the *local GN*, (2) unique alleles from the *external GN* or (3) a combination of both, depending on the selected strategy (see *Provenancing Scenarios* section). To ensure that unique diversity from the *external GN* was effectively captured, alleles identified as common within the *local GN* were first removed from the *external GN* dataset. The MAF was then recalculated, and alleles remaining in the *external GN* matrix (i.e., not common in the *local GN*) were classified as either uniquely common or rare among samples within the *external GN*. This approach maximised the likelihood of incorporating novel alleles from the *external GN* rather than duplicating those already present locally.

### 2.4 Step 2: Practitioner Input Decisions to Establish Representative Diversity in SPAs

To establish representative SPAs while accounting for real‐world constraints in the provenancing germplasm material, practitioners are given flexibility to make three key decisions that shape the overall sampling strategy.

#### 2.4.1 Decision 1: Minimise Total Individuals or Minimise Sites to Sample

Practitioners must choose between two mutually exclusive approaches: (1) minimising the total number of individuals to be planted in the SPA or (2) minimising the number of in situ sites that must be accessed to collect material. This decision directly influences the sampling strategy and determines which combinations are considered in downstream analyses. These approaches are conceptually similar to the evenly dispersed versus clustered sampling patterns proposed by Marshall and Brown (1975). A trade‐off exists between the two: minimising individuals typically requires sampling from more sites to capture sufficient common allelic diversity, whereas minimising sites often requires sampling more individuals per site to achieve comparable genetic representation.

To determine the minimum number of samples and sites required to capture a target proportion of common alleles within the focal GN, we implemented a random sampling procedure. In this process, a specified number of individuals or sites were randomly selected across the GN, and the proportion of common alleles captured within each subset was calculated. For each sample size, 10,000 randomisations were performed, and the smallest number of individuals or sites (depending on practitioner preference) was identified, where all iterations captured > 90% of the common alleles present in the target GN. This 90% threshold follows Díaz et al. (2020), who recommend retaining > 90% of wild intraspecific genetic diversity. Although our focus is on capturing evolutionary potential within newly established SPAs rather than maintaining in situ diversity, this benchmark provides a practical, quantifiable target for guiding genetic representation in ex situ collections. However, the threshold can be adjusted to align with specific restoration objectives if needed.

For individual‐based randomisations, sample sizes ranged from 1 to 30 individuals. Progressive sampling began with one individual per site, increasing to two per site after reaching the dataset's maximum number of sites, and so on. For site‐based randomisations, sample sizes ranged from 1 to 10 sites. In each case, five individuals were randomly sampled per site, resulting in total sample sizes from 5 to 50 individuals (i.e., 1–10 sites × 5 individuals per site).

#### 2.4.2 Decision 2: Unconstrained or Constrained Sampling

While sampling small amounts of leaf tissue for an initial genomics study can be relatively straightforward across many sites and species, restoration practitioners collecting larger amounts of germplasm material often face post hoc logistical limitations that restrict which sites or individuals can be sampled, thereby influencing the final sampling strategy. Practitioners may choose to include or exclude specific sites or individuals due to factors such as restricted access (e.g., geopolitical boundaries or changes in permit limitations), the availability of existing material already held in ex situ collections (e.g., existing nursery stock) or logistical convenience (e.g., accessibility and ease of sampling at a known site).

For this study, we created a hypothetical list of sites from both the *local* and *external GN*s to be either excluded or included across each provenancing scenario (Table S2 in Appendix 3). These sites (and all associated individuals within each site) represent a *constrained* sampling approach, where provenancing scenarios aim to maximise allelic representation within predefined limitations. This constrained scenario serves as one illustrative example among many possible constrained sampling configurations incorporating forced inclusion or exclusion rules. Restoration practitioners may therefore approach each provenancing scenario as either: (1) *Unconstrained*: with full freedom to sample any site or individual across the GN, or (2) *Constrained*: with specific site‐ or individual‐level restrictions based on real‐world considerations.

#### 2.4.3 Decision 3: Random or Optimised Sampling

In this final decision, practitioners select whether to undertake random or optimised sampling across the target GN. The *random* approach allows any combination of individuals or sites to be selected within the defined area (following the previous practitioner‐defined constraints). While selections are random, the strategy still aims to capture > 90% of common alleles of the focal GN within the ex situ collection. The *optimised* approach, by contrast, identifies a specific fixed combination of individuals or sites that maximises genetic diversity, generally achieving a substantially higher representation of common alleles but at the expense of flexibility. In practice, the randomised strategy provides more operational freedom, whereas the optimised strategy enhances allelic representation but is less adaptable.

To maximise the representation of common alleles within each target GN, we implemented a simulated annealing optimisation (Kirkpatrick et al. 1983), which uses a genetic diversity measure based on a down‐projected site frequency spectrum (SFS). This approach was adapted from the OptGenMix framework, originally using *Nei* diversity as the metric to be optimised (Bragg et al. 2020), instead modified here to optimise both individual and site combinations.

Our diversity metric, termed *psfs*, quantifies the proportion of non‐fixed loci after down‐projecting a stacked SFS to a fixed sample size (*m*), following Marth et al. (2004) and Exposito‐Alonso et al. (2022). This down‐projection allows fair comparison across datasets with varying degrees of missing data and serves as a robust analogue of polymorphic‐locus counts. Details of *psfs* calculation are provided in Appendix 4, with illustrative examples for both individuals and sites presented in Figures S7 and S8.

The simulated annealing algorithm searched for combinations of individuals or sites that maximised *psfs*. The optimisation iteratively explored alternative combinations, occasionally accepting suboptimal solutions to avoid local maxima, and converged on an approximate global optimum. Optimisations were conducted in R using modified versions of the *OptGenMix* (https://github.com/jasongbragg/OptGenMix/tree/sfs) and *sfsCalcs* (https://github.com/jasongbragg/sfsCalcs) packages. Constrained optimisations, where specific samples or sites were required to be included or excluded, were implemented by fixing their weights within the optimisation process. Detailed descriptions of R functions, parameter settings and implementation details regarding these optimisations are outlined in Appendix 4.

Convergence of each optimisation was verified by inspecting temperature profiles and plateau behaviour across 10,000 simulated annealing steps. The performance of optimised selections was then evaluated against results from random sampling (see Decision 1: Minimise total individuals or minimise sites to sample), using the proportion of common and rare alleles captured as a proxy for *psfs* optimisation success.

### 2.5 Step 3: Provenancing Scenarios

Based on the three practitioner‐defined decisions outlined above, we demonstrate how our workflow can be applied to three key provenancing scenarios (A, B and C) for establishing genetically representative SPAs. Table S1 in Appendix 3 links each applied scenario to commonly used terms in the literature, providing both simplified and applied examples corresponding to each strategy. For constrained sampling options (as described in Decision 2 above), we implement forced inclusion and exclusion of specific sites (Table S2 in Appendix 3), where applicable for each scenario. Accordingly, any individuals located within the specified sites are either included or excluded from the sampling pool, depending on the constraint applied.

#### 2.5.1 Scenario A—Local Provenancing

This scenario guides representative provenancing within the identified *local GN* only. This scenario is considered a form of broad local provenancing or a localised form of admixture/composite provenancing (Harrison et al. 2021; Prober et al. 2015). In this study, the focus is solely on capturing representative diversity across the entire *local GN*. While similar to regional admixture provenancing as described by Bucharova et al. (2019), our approach differs in that material is sourced based on regions of genetic structure (GNs), rather than areas defined by environmental similarity. This allows sampling across biogeographical areas where historical gene flow and genetic structure connect sites.

#### 2.5.2 Scenario B—Predictive Provenancing

This scenario focuses on representative provenancing of genetic material unique to the *external GN* only. It aligns with the concept of predictive provenancing described by Prober et al. (2015). Common alleles already present in the *local GN* are excluded (see Allele classification for *local* and *external GN*s), ensuring that the scenario sources material solely from the *external GN*. The aim is to introduce genetic diversity from areas expected to match future climate conditions, without attempting to retain representative local diversity in the final planting design.

#### 2.5.3 Scenario C—Climate‐Adjusted Provenancing

This scenario provides guidance for representative provenancing that includes both local genetic material (representative of the *local GN*) and climate‐resilient material from the *external GN*. In essence, it combines the approaches outlined in Scenarios A and B, incorporating both local diversity and putative future climate‐adapted diversity (Harrison et al. 2021). This strategy aligns with concepts of climate‐adjusted, admixture and composite provenancing as defined by Prober et al. (2015), but applies them using defined *local* and *external GN*s, rather than relying on a continuous geographic distance gradient. Unlike predictive provenancing, which does not consider representing local diversity, climate‐adjusted provenancing integrates both *local* and external sources, enhancing both genetic representation and climate resilience in final planting.

The workflow presented in Figure 2 outlines the key steps in designing a germplasm collection strategy to capture representative genetic diversity from both *local* and *external GN*s and demonstrates how these steps align with major germplasm sourcing strategies described in the literature (i.e., local, predictive and climate‐adjusted; see Table S1 in Appendix 3).

### 2.6 Validation Testing of Sampling Outcomes

We wanted to evaluate how flexible changes to both the random and optimised outcomes in this study may be, particularly in the context of potential adjustments to the sampling design that may occur during germplasm collection, propagation and SPA establishment. To do this, we performed three validation tests related to provenancing Scenario A, quantifying allele capture under the following conditions: (1) removal of samples from the final optimised combinations, (2) the impact of site removal on random sampling within the *local GN* dataset and (3) alternative sampling guidelines for fixed (optimised) combinations. Detailed methods for these validation tests are provided in Appendix 5.

## 3 Results

**FIGURE 3:**
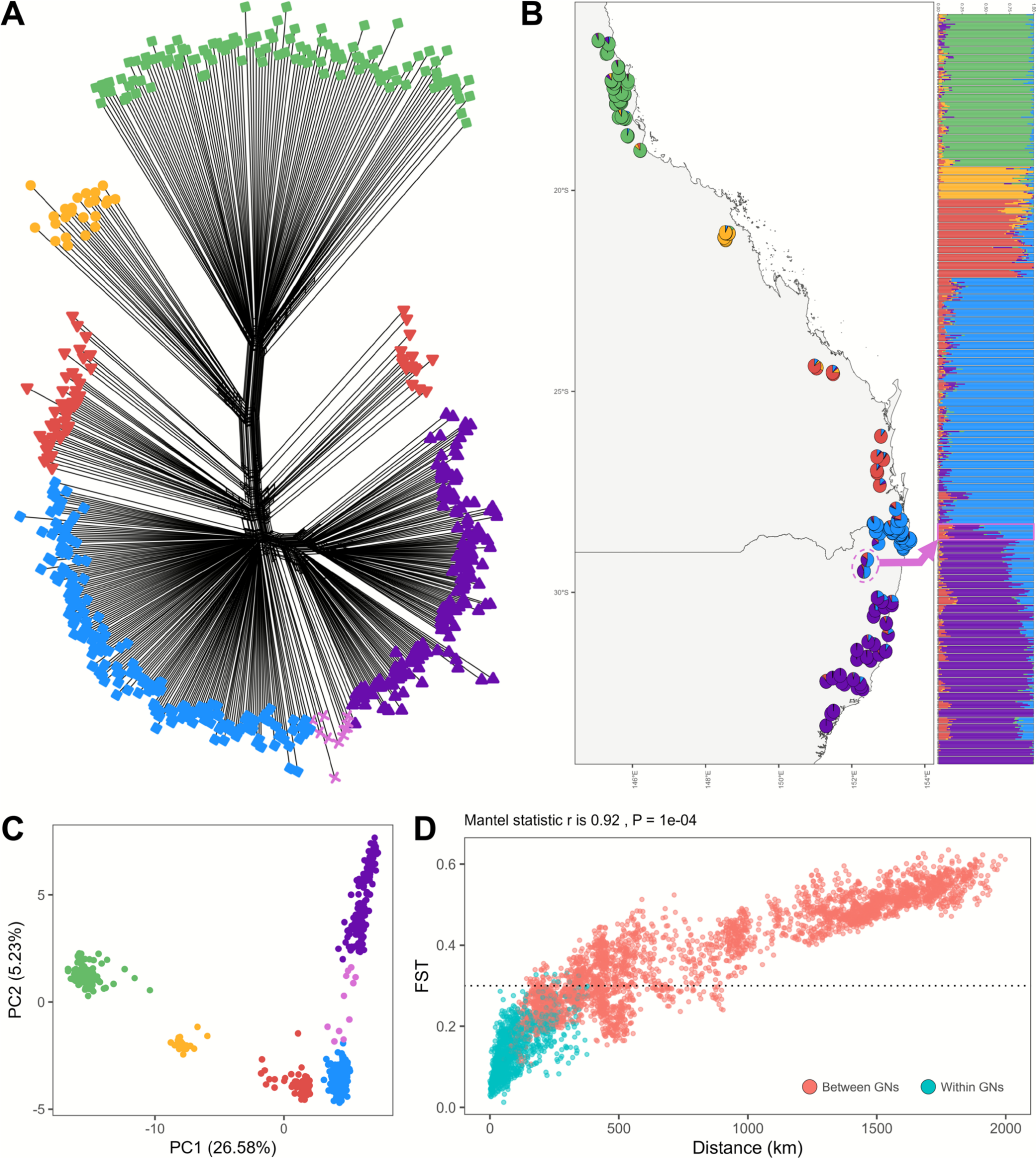
Population genetic analyses highlighting the genetic neighbourhoods (GNs) across the extant distribution of *Neolitsea* dealbata. (A) Splitstree network with nodes coloured by GN. (B) LEA sNMF pie chart and bar plots of K = 5, with a zone of admixture highlighted (pink arrow). (C) Principal component analysis (PCA) of individuals coloured by GN. (D) Isolation by distance (IBD) plot coloured by pairwise comparisons between GNs (coral) and within GNs (teal). (including the zone of admixture), with significant mantel statistic provided.

A total of 554 individuals across 95 sites were successfully sequenced spanning the extant distribution of *N. dealbata* , yielding 14,344 filtered SNPs to identify GNs. We identified five GNs and one zone of admixture across the species' range, reflecting an overall pattern of IBD along its latitudinal distribution (Figure 3). The identified *local* and *external GN*s (Figure 1) showed clear geographic and genetic differentiation from each other, while maintaining population pairwise F_ST_ values < 0.5 (Figure S6 in Appendix 2). The filtered SNP data resulted in 14,344 SNPs for the entire dataset, 6132 SNPs for the *local GN* dataset (1200 common and 4932 rare alleles) and 5039 SNPs for the *external GN* dataset, with 4152 of these representing alleles that are not classified as common in the *local GN* (1004 common and 3148 rare alleles).

**FIGURE 4:**
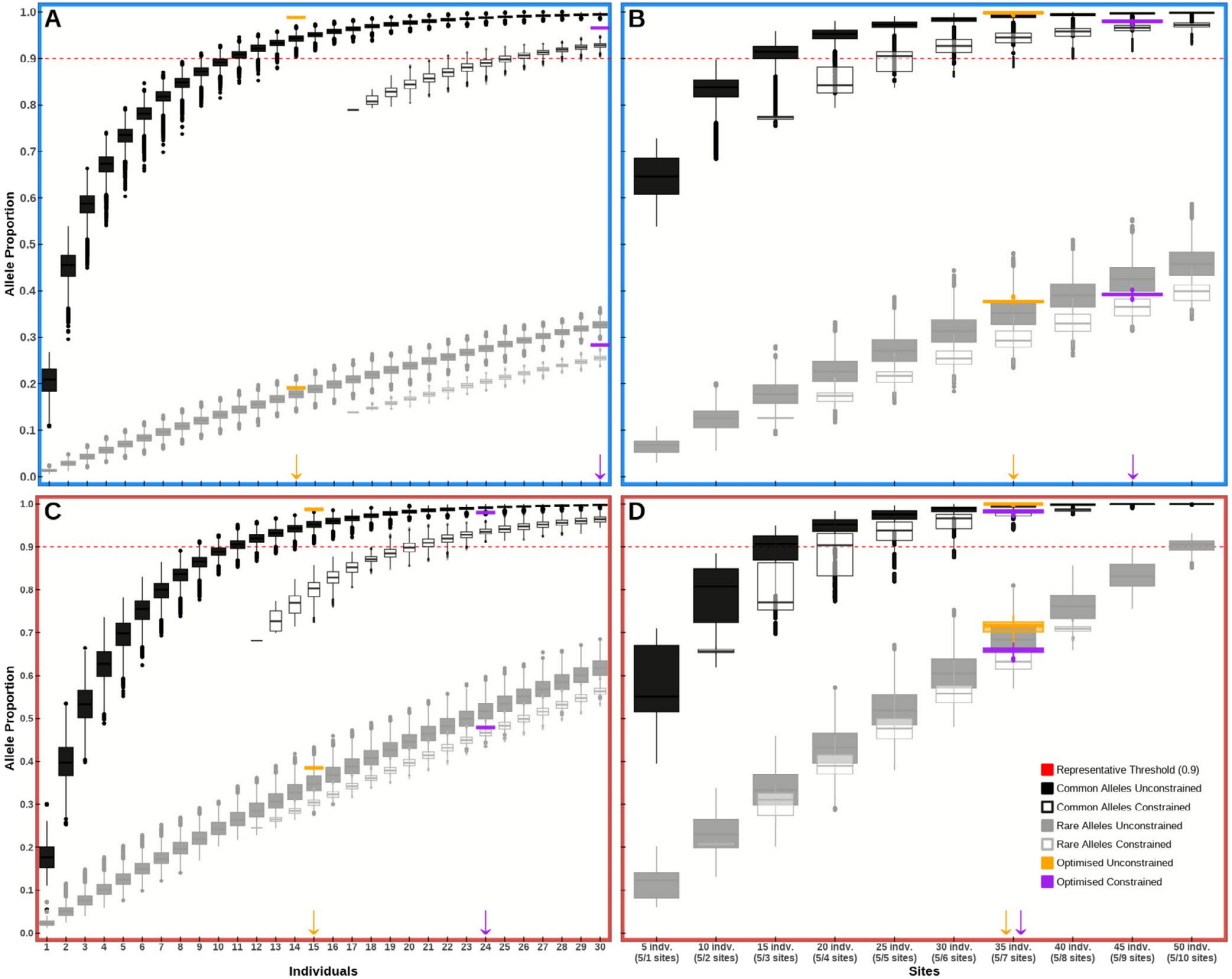
Proportion of common (black) and rare (grey) alleles captured across randomised sampling combinations for both the *local GN* (blue border; A and B) and unique alleles found in the *external GN* (red border; C and D). Sampling approaches reflect alternative practitioner‐led decisions: Decision 1: Minimising either the total number of individuals (A and C) or the total number of sites (B and D); Decision 2: Filled boxplots represent unconstrained sampling, while unfilled boxplots represent constrained sampling (see constraints in Table S2 in Appendix 3); Decision 3: For each case, the random sampling combination capturing > 90% of common alleles with the smallest number of individuals or sites is indicated with an arrow. Allele proportions of optimised combinations are shown in orange or purple, either as fixed lines or as boxplots when variation arises from which individuals are represented for site combinations.

In each scenario, random sampling determined the minimum number of individuals or sites needed to represent 90% of common alleles under both unconstrained and constrained conditions (Figure 4; Table 1). All optimised strategies captured an equal or greater proportion of common alleles compared to the maximum value observed in random sampling (Figure 4). Although optimisation focused solely on common alleles, these strategies generally represented a higher proportion of rare alleles than most random samples, as illustrated in the boxplot comparisons.

**TABLE 1:** Sampling strategies and common allele representation for provenancing scenarios A and B across all possible practitioner‐defined decisions. Bold text signifies forced sampling combinations for constrained scenarios (Table S2 in Appendix 3).

Scenario	Practitioner input			Sampling strategy	Total individuals	Total sites	Common allele representation
	Decision 1	Decision 2	Decision 3				
Scenario A (*Local GN*)	Minimise individuals	Unconstrained	Randomised	Any 14 individuals (1 individual/site)	14	14	90.42%–97.00%
			Optimised	Specific 14 individuals	14	13	98.83%
		Constrained	Randomised	**17 forced individuals** + Any 13 individuals (1–2 individuals/site)	17 + 13	3 + 11	90.33%–94.92%
			Optimised	**17 forced individuals** + Specific 13 individuals	17 + 13	3 + 4	96.58%
	Minimise sites	Unconstrained	Randomised	Any 5 individuals/site across any 7 sites	35	7	91.17%–100%
			Optimised	Any 5 individuals/site across specific 7 sites	35	7	99.33%–100%
		Constrained	Randomised	**3 forced sites (5 individuals/site)** + Any 5 individuals/site across any 6 additional sites	15 + 30	3 + 6	91.67%–98.67%
			Optimised	**3 forced sites (5 individuals/site)** + Any 5 individuals/site across specific 6 additional sites	15 + 30	3 + 6	97.17%–98.50%
Scenario B (*External GN*)	Minimise individuals	Unconstrained	Randomised	Any 15 individuals (1–2 individuals/site)	15	10	90.74%–98.12%
			Optimised	Specific 15 individuals	15	9	98.71%
		Constrained	Randomised	**12 forced individuals** + Any 12 individuals (1–2 individuals per site)	12 + 12	2 + 8	91.14%–96.12%
			Optimised	**12 forced individuals** + Specific 12 individuals	12 + 12	2 + 5	98.80%
	Minimise sites	Unconstrained	Randomised	Any 5 individuals/site across any 7 sites	35	7	94.12%–100%
			Optimised	Any 5 individuals/site across specific 7 sites	35	7	99.80%–100%
		Constrained	Randomised	**2 forced sites (5 individuals per site)** + Any 5 individuals/site across any 5 additional sites	10 + 25	2 + 5	93.82%–98.90%
			Optimised	**2 forced sites (5 individuals per site)** + Any 5 individuals/site across specific 5 additional sites	10 + 25	2 + 5	97.11%–98.80%

*Note:* For each case, the minimum number of sites to sample from is provided, based on the total number of available sites in the dataset under both unconstrained and constrained decisions (see constraints in Table S2 in Appendix 3). To implement the climate‐adjusted provenancing Scenario C described in this study, select one strategy from Scenario A and one from Scenario B as desired.

Temperature plots for each optimisation indicated a global optimum reached for each analysis (Figure S9 in Appendix 4). The geographic distribution of these optimised combinations is shown in Figure S10 in Appendix 4, demonstrating relatively even spatial coverage across available sites within each scenario. Unlike random sampling, which assumes one individual per site, the optimisation analyses for minimising individuals allowed for sampling of multiple individuals per site. Consequently, optimised combinations sometimes included fewer total sites than random sampling (Table 1). As shown in Table 1, individual‐based optimised combinations have a fixed allele proportion, whereas site‐based optimisation strategies exhibit a range of allele proportions. This variation arises because sampling within fixed site combinations is limited to five samples per site, though some sites may have six individuals in the dataset.

The frequency of common alleles represented by each sample in the individual‐based optimised outcome for the *local GN* is visualised in Figure S11 in Appendix 4. The number of common alleles represented by less than 10% of optimised samples was 9.58% and 16% for the unconstrained and constrained outcomes, respectively. However, more common alleles were represented by only 1–5 samples in the unconstrained optimised combination, likely due to the substantially smaller number of individuals compared to the constrained combination (14 vs. 30 individuals).

### 3.1 Representative Diversity for Scenario A

Following the unconstrained sampling option, the minimum combination required to randomly represent more than 90% of common alleles in the *local GN* (Scenario A) is either 14 individuals (one individual per site across 14 sites) or 35 individuals (five individuals per site across seven sites; Figure 4A,B, Table 1). Under the constrained option, achieving the same target requires 30 individuals, including 17 pre‐selected individuals plus 13 additional individuals (sampled from one to two per site across 11 additional sites) or 45 individuals, including five individuals from each of three pre‐selected sites plus five individuals from six additional sites.

These results demonstrate that representative diversity can still be achieved under a highly constrained scenario, but additional sampling is necessary to capture the same proportion of common alleles across the GN. They also indicate that a historical “local‐is‐best” approach to provenancing would fail to capture 90% of common alleles. Sampling a single individual from the *local GN* captures less than 30% of common alleles, while sampling a single site (five individuals) captures less than 75%. Even a more relaxed approach, sampling two sites at random with five individuals per site, still fails to reach the 90% threshold (Figure 4B).

### 3.2 Representative Diversity for Scenario B

Under the unconstrained option, the minimum sampling combination required to randomly capture more than 90% of common alleles from the *external GN* that are not common in the *local GN* is either 15 individuals (one to two individuals per site across 10 sites) or 35 individuals (five individuals per site across seven sites; Figure 4C,D, Table 1).

For the constrained option, achieving the same target requires 24 individuals, including 12 pre‐selected individuals plus 12 additional individuals (sampled one to two per site across eight additional sites) or 35 individuals, including five individuals from each of two pre‐selected sites plus five individuals from five additional sites.

As observed for the *local GN*, a “local‐is‐best” approach sampling only one individual or one site (five individuals) fails to capture 90% of uniquely common alleles from the *external GN*, representing less than 30% and 75% of common alleles, respectively.

### 3.3 Representative Diversity for Scenario C

To implement the sampling strategy for provenancing Scenario C, any combination of outcomes from Scenarios A and B (Table 1) can be combined to capture representative diversity across both the local and *external GN*. This approach allows flexibility in applying different practitioner‐defined decisions for each scenario. For example, one could choose an optimised, constrained, individual‐minimising strategy for the local GN while using a randomised, unconstrained, site‐minimising approach for the *external GN*. Regardless of the combination selected, the final planting consistently captures over 90% of common alleles from the *local GN* and over 90% of unique common alleles from *the external GN* (i.e., alleles not common in the *local GN*), ensuring comprehensive genetic representation.

### 3.4 Validation Testing of Sampling Outcomes for Scenario A

#### 3.4.1 Test 1: Impact of Sample Removal on Allelic Representation

Results from Test 1 showed that randomly removing 1–5 individuals from the optimised combinations generated for Scenario A led to a decrease in both common and rare alleles represented (Figure S12 in Appendix 5). While the removal of 1–4 individuals from the unconstrained outcome may still represent 90% of common alleles for the *local GN*, the removal of 5 samples may not. This indicates that mortality during and after planting should be supplemented to maintain genetic representativeness. For the constrained outcome, the larger total number of plants (30 individuals) provides a buffer, such that the removal of any 5 individuals from the optimised combination still represents more than 90% of common alleles within the *local GN*.

#### 3.4.2 Test 2: Effects of Site Removal on Sampling Outcomes

For Test 2, the removal of sites from the *local GN* does not alter the number of samples required to represent common allelic diversity, provided that geographic representation across the *local GN* is maintained. However, outcomes are affected if site removal introduces substantial geographic bias (Figure S13 in Appendix 5). Therefore, capturing representative diversity of common alleles is robust to variation in sampling density, as long as sites across the species' extant range (and consequently the target GNs) are included. If significant geographic regions are absent from the dataset, the capture of common allele diversity may be biased, leading in the final plantings.

#### 3.4.3 Test 3: Consequences of Replacing Optimised Individuals

In Test 3, replacing individuals from the optimised combination with alternative individuals from the same site still captured more than 90% of common alleles across the *local GN* for both unconstrained and constrained options (Figure S14 in Appendix 5). Although the capture of common alleles is slightly reduced when optimised individuals are replaced, sufficient genetic diversity is maintained, allowing flexibility if specific individuals at a site cannot be sampled.

## 4 Discussion

Higher intraspecific diversity within populations generally enhances the overall adaptive capacity to biotic and abiotic challenges, thereby increasing overall fitness (Reed and Frankham 2003). In this study, we present strategies for developing representative germplasm collections for SPAs that maximise adaptive potential while accommodating practitioner‐driven decision‐making during SPA design. By following established provenancing approaches regarding local, predictive and climate‐adjusted provenancing (Table S1), the workflow facilitates the production of seed collections that capture broader individual‐level variation (Clark 2010) and enhance adaptive capacity under climatic extremes (Reusch et al. 2005).

In many systems, neutral genetic variation often reflects much of the total genomic diversity, with patterns of population structure and adaptive potential showing strong concordance whether based on all, neutral or adaptive markers (Batista et al. 2016; Chung et al. 2023; Fitzpatrick et al. 2021; Lind and Lotterhos 2025). Accordingly, by quantifying allelic representation across all available SNPs, rather than separating putatively neutral or adaptive loci, our analyses capture broad genomic patterns that serve as a reliable proxy for adaptive capacity in SPA design.

The workflow presented here can be applied for sampling a diverse ex situ collection for any species that comprise geographically partitioned GNs across their extant range. Using GNs to guide provenancing germplasm collections allows this workflow to be implemented across a spectrum from narrow‐range endemics to broadly distributed common species, ranging from species distributions of several hundred meters to several hundred kilometres. This approach helps resolve issues discussed in previous studies (Breed et al. 2018; Broadhurst et al. 2023), regarding the need to define the geographical extent of local provenances, and provides a method for how non‐local provenances can be incorporated into SPAs for a local target restoration area.

However, as noted by Fahey et al. (2025), discrete GNs may not always be identifiable due to factors such as ploidy variation, mating systems or hybridisation. In these cases, a more bespoke approach, targeting specific individuals or genetically screening candidate plants, may be required. For species characterised by a single, largely undifferentiated GN, applying *F*_ST_ based isolation‐by‐distance thresholds may be more suited for identifying meaningful sampling regions (e.g., Rossetto et al. 2019). In both scenarios, quantifying allele capture and optimising sampling combinations remain valuable for guiding collection design.

In this study, GN assignment was excluded for a zone of admixture (Figure 3). Hypothetically, if the target restoration site were located within this zone, the local GN should encompass both ancestral sources, representing multiple “local” GNs. For instance, the admixed (Washpool) area in Figure 1 would incorporate “Northern NSW” and “Southern NSW” as *local* GNs, with “South‐East Queensland” as an *external GN*. This approach ensures the representation of common alleles across the local region while integrating unique alleles from external sources.

### 4.1 The Importance of Quantifying the Scale of ‘Local’ Provenancing

Our results reinforce growing evidence that strict local provenancing (in this case referred to as sampling from one individual or one site of 5 individuals) fails to represent sufficient genetic diversity within ex situ collections, leading to genetically depauperate plantings which are compounded further in fragmented landscapes (Breed et al. 2018; De Vitis et al. 2022; Jordan et al. 2019). The geographic scale of our identified GNs, spanning 100's of kilometres rather than 100's of metres, confirms that genetic turnover (and thus local provenancing boundaries) operates at far broader spatial scales than traditionally assumed (Fahey et al. 2024). This supports critiques of the “local‐is‐best” paradigm (Broadhurst et al. 2023) and argues for defining local provenance boundaries based on genetic structure rather than geographic proximity or community identity.

### 4.2 Integrating Local Representation With Future Resilience

Consistent with previous recommendations (Breed et al. 2013; Prober et al. 2019; Sgrò et al. 2011), enhancing the adaptive capacity of ex situ collections may require incorporating genotypes from beyond the local area to ensure long‐term population viability under environmental stress. Sourcing material from non‐local, heat‐tolerant environments has increased climate resilience in both marine (Morikawa and Palumbi 2019) and terrestrial (Nolan et al. 2023) ecosystems, though some studies report limited benefits from warmer‐climate provenances (Bjorkman et al. 2017; Hancock and Hughes 2014).

Our approach offers a balanced framework; by sampling representatively across entire GNs that overlap with climate‐matched areas, practitioners can capture broad genetic variation (including potentially adaptive diversity) without necessarily restricting sourcing to specific sites. This GN‐wide strategy shifts the focus from population‐level to neighbourhood‐level adaptation, capturing a broader pool of common alleles. In our study, the *external GN* contributed 1004 unique common alleles, rare or absent in the local GN, effectively doubling the available diversity for SPA design.

Although we provide a predictive provenancing example (Scenario B), we believe designing SPAs solely around climate‐resilient material should not be the exclusive goal (as outlined in Broadhurst et al. 2023). A climate‐adjusted provenancing approach that balances local genetic representation with future resilience remains optimal.

While we are assuming some degree of compatibility for co‐planting of both local and external genotypes in the SPA, the relatively close geography and low F_ST_ values between the two GNs suggest that potential risks of maladaptation and outbreeding depression are minimal. Even when outbreeding occurs between divergent populations, negative effects often diminish over subsequent generations (Erickson and Fenster 2006). Thus, integrating external genetic material into a diverse local SPA is likely to yield a net positive effect. Maximising overall genetic diversity promotes adaptive potential through diversification and heterosis, enhancing population persistence under future climatic extremes. However, where no climate‐matched *external GN* exists, sourcing from adjacent or environmentally similar regions/GNs should still be considered to broaden genetic diversity and adaptive capacity.

### 4.3 Trade‐Offs for Maximising Genetic Representation With Practical Considerations

Several stages of the proposed workflow incorporate practitioner input into the final sourcing strategy. The preferred option will depend on project‐specific constraints such as budget, nursery capacity, site accessibility and concurrent sampling for other species.

For Decision 1, reducing the number of representatives per germplasm line can lower propagation and housing demands but requires a broader spatial sampling effort across the target GN. Conversely, reducing the total number of sites simplifies logistics yet increases propagation and planting loads.

For Decision 2, applying constraints, whether forced inclusion or exclusion of sites/individuals, typically requires sampling more material to achieve comparable genetic representation. Even so, GN‐wide diversity can still be maintained. In some cases, forced inclusion of known genotypes already held in nurseries or ex situ collections may reduce the need for additional in situ sampling.

For Decision 3, optimised sampling strategies identify combinations of sites or individuals that maximise common‐allele capture and outperform random sampling in representing allelic variation. These optimised outcomes also allow integration with multi‐objective frameworks, such as that of Bragg et al. (2020), which maximise *Nei's* diversity while minimising mean pairwise kinship, a useful approach when several individuals occur per site. In contrast, randomised sampling offers greater flexibility, particularly when collecting multiple species simultaneously. Allowing practitioners to select any site within a GN enables overlapping collections across species, reducing the total number of sites visited and the overall sampling effort for multi‐species projects.

### 4.4 Type and Traceability of Restoration Material

The workflow presented here can be applied to a range of germplasm types, including cuttings, seedlings and in situ seedling banks. When sampling multiple individuals from a site, best practice is to maintain separate maternal lines, regardless of material type, to preserve genetic representation (van der Merwe et al. 2023).

It is not always possible to use the exact individuals that were genotyped in the original dataset. For seeds or in situ seedlings, a one‐to‐one correspondence with genotyped plants rarely exists unless downstream genetic screening is undertaken. Similarly, cutting material collected from untagged individuals or from new sites within the target GN may lack known maternal lineages. In such cases, we recommend (1) prioritising sites with low clonality or kinship where such data are available and (2) ensuring spatially distributed sampling across the population to reduce the likelihood of collecting genetically identical ramets.

For long‐lived species such as *N. dealbata* , it may take decades for an SPA to yield large quantities of genetically diverse seed. However, establishing genetically diverse plantings early enables cuttings to be collected from immature saplings or small trees, allowing material to be used immediately in current restoration efforts across the target area. This approach minimises repeated in situ sampling while facilitating the replication of representative material across multiple sites within the GN.

### 4.5 Scaling Up or Down the Total Individuals in the SPA

Our workflow sets minimum thresholds for individuals and sites needed to represent common alleles within a GN, but SPAs may sometimes involve hundreds to thousands of plants to meet high seed yield demands. While smaller numbers suffice to capture common allelic diversity, larger plantings require a scalable approach.

Increasing total individuals can be achieved by adding more sites across the focal GN, which maintains high allelic diversity and low kinship, though this can be increasingly impractical for very large plantings. Alternatively, increasing the number of individuals per site proportionally allows scalable plantings while keeping site representation balanced. However, developing large plantings using a relatively small number of sites is likely to have it's own challenges. Therefore, we recommend future studies evaluate how proportional scaling affects both allelic representation and kinship.

Conversely, some ex situ collections may require less than the minimum suggested individuals provided in the workflow, i.e., scaling down the total number of individuals. In such cases, the *psfs* optimisation allows selection of a fixed subset of individuals while preserving maximum allelic coverage, providing flexibility when practical constraints limit the overall collection size.

### 4.6 Broader Applications

The workflow presented here provides a structured approach for establishing SPAs within a target restoration region (e.g., the Big Scrub lowland subtropical rainforest), but its utility extends well beyond this context. It can also be applied to safeguard genetic diversity in ex situ germplasm banks and living collections in botanic gardens, to supplement existing collections to meet allelic representation thresholds, and to guide the establishment of new in situ populations in previously cleared areas.

Inadequate diversity in ex situ holdings remains a persistent issue. Genomic screening of existing collections can identify underrepresented lineages or regions that require supplementation (Diniz‐Filho et al. 2012; Murrell et al. 2025). Our workflow can integrate existing ex situ material through forced inclusion, then identify genetically complementary wild individuals/sites to enhance overall diversity. This approach may also support small‐population supplementation when the complete genetic composition of an existing site is known, although scaling it to large, complex populations warrants further study (see also *Scaling up or down the total individuals in the SPA*).

While this study focuses on representative common allelic diversity across all SNPs, the workflow could be adapted to target putatively adaptive alleles linked to climate or functional traits (e.g., Aitken et al. 2024; Breed et al. 2019; Carvalho et al. 2021). Incorporating key adaptive alleles via forced inclusion, then complementing with broad GN‐wide allelic coverage, would promote both trait‐specific adaptation and overall genetic resilience. Multi‐objective optimisation frameworks, such as Bragg et al. (2020), allow the inclusion or exclusion of specific alleles or traits when designing translocation plantings.

Finally, sourcing material across multiple populations within climatic seed transfer zones (regional admixture provenancing) is a growing strategy (Bucharova et al. 2019). Our GN‐focused workflow could potentially integrate climate‐matched zones, broadening its applicability for regional and global restoration strategies.

## 5 Conclusion

We present a flexible, genetically informed workflow that guides species‐specific SPA design while also being able to be replicated across multiple species at a time with overlapping GNs. By offering a variety of provenancing approaches from optimised combinations to more flexible, randomised sampling, the workflow supports practitioners in designing tailored, evidence‐based strategies that can align with specific project goals and logistical realities.

## Author Contributions

**Richard J. Dimon:** conceptualization (equal), data curation (equal), formal analysis (lead), investigation (lead), methodology (equal), writing – original draft (lead), writing – review and editing (equal). **Jason Bragg:** conceptualization (equal), formal analysis (supporting), methodology (equal), writing – review and editing (equal). **Patrick Fahey:** conceptualization (equal), validation (equal), writing – review and editing (equal). **Marlien van der Merwe:** conceptualization (equal), validation (equal), writing – review and editing (equal). **Peter D. Wilson:** conceptualization (equal), formal analysis (supporting), methodology (equal), writing – review and editing (equal). **Robert Henry:** project administration (supporting), supervision (equal), writing – review and editing (equal). **Maurizio Rossetto:** conceptualization (equal), funding acquisition (lead), project administration (lead), supervision (equal), writing – review and editing (equal).

## Funding

This research was partly funded by the NSW Government Environmental Trust as part of the Science Saving Rainforests project. Relevant Environmental Trust grants relating to this project were awarded to the Big Scrub Rainforest Conservancy (2019/RD/0006 and 2019/RR/0098) and the Big Scrub Foundation (2020/RR/0105).

## Conflicts of Interest

The authors declare no conflicts of interest.

## Supporting information


**Appendix S1–S4:** ece372658‐sup‐0001‐supinfo.docx.

## Data Availability

Output of SNP data of *local* and *external GN*s in this paper and corresponding scripts have been deposited in the University of Queensland's eSpace data repository under accession code UQ:1b70ddb (https://espace.library.uq.edu.au/view/UQ:1b70ddb). Detailed results from each analysis are provided in Supporting Information.
