# Supplementary material for: Integrating Genomic and Climate Data to Design Representative Seed Production Areas: A Pragmatic Workflow for Climate‐Adjusted Provenancing
Source: Ecol Evol. 2026 Jan 15;16(1):e72658. doi: 10.1002/ece3.72658 (PMC12808335; doi:10.1002/ece3.72658)
Supplement: Supplementary file 1 — Appendix S1–S4: ece372658‐sup‐0001‐supinfo.docx. [file ECE3-16-e72658-s001.docx]

**Appendix 1: Site and Regional Matching, and Environmental Variable Selection**

*Site matching principles*

Site matching has long been used in forestry and restoration ecology (Broadmeadow et al. 2005; Harrison et al. 2017; Rossetto et al. 2019; St. Clair et al. 2022) to increase the likelihood that selected propagation material has experienced environmental conditions broadly similar to those of the target site. Consequently, the material is more likely to have undergone comparable local adaptive and other genetic processes.

Key considerations in applying site matching include the selection of environmental variables, the choice of climate change scenario and time period, and the matching method. This appendix summarises the decisions made in developing an updated regional site-matching approach for the *Restore and Renew* webtool.

*Variable selection*

Selecting variables is a fundamental first step in any attempt to characterise the relationship between biological entities (e.g. species occurrences), their attributes (e.g. allele frequencies and spatial distributions), and the environments in which they occur, or which are believed to have driven processes such as local adaptation and other population genetic processes (Williams et al. 2012).

A small set of variables was chosen when developing site matching for the Restore and Renew webtool (Rossetto et al. 2019), since they were considered sufficient to describe the environmental characteristics of a restoration site and to represent environmental attributes hypothesised to influence population and genetic processes. The selected variables were:

- Mean annual temperature: Characterises the basic thermal environment
- Mean annual rainfall: Characterises the overall availability of moisture
- Temperature seasonality: Describes the seasonal temperature contrast
- Precipitation seasonality: Describes the seasonal pattern of moisture availability
- Aspect: Influences the intensity of insolation
- Topographic wetness index: Indicates the way soil moisture may move through a site

It is critical that all selected variables are available over the entire extent of interest and that the quality of mapped variables is sufficient to support their use in site matching. This may mean that important environmental characteristics must excluded from site matching. For example, when developing the *Restore and Renew* webtool, we were aware of the fundamental importance of soil characteristics to plant species. However, we concluded that available soil mapping was not reliable enough, and was not at a sufficient spatial grid resolution, to use with other environmental layers.

To allow for critical but unavailable variables to be included in final planning for restoration, *Restore and Renew* webtool users are advised to use local knowledge of other environmental features including soil types to focus on potential collection sites within the broad environmental matches output by the webtool.

Finally, a fundamental constraint on variable selection for site matching is the “curse of dimensionality”. This refers to the phenomenon observed in multivariate analyses where adding additional variables progressively constrains the spatial region which matches a focal site. This may lead to implausibly small and highly disjointed matching regions. Figures S1-S3 inclusive illustrate this phenomenon using five of the six variables available in the webtool.

*Future climate*

Including future climate projections requires two additional choices: the climate change scenario and the future time period. The Restore and Renew webtool uses data downscaled from the Fifth Assessment Report of the Intergovernmental Panel on Climate Change (IPCC). These data allow users to select between a “moderate” scenario (RCP4.5) and a “severe” scenario (RCP8.5).

Climate model outputs are available for present conditions through to 2100, allowing the generation of climate averages for variables such as mean annual temperature over any chosen period (typically 10–30 years). For the Restore and Renew webtool, we selected two 30-year climate averages centred on 2050 and 2070. In this study, only 2070 modelled future climate is presented.

*Current and future matching*

Matching to current climate is undertaken using observed climate layers. Future climate matching, used to implement assisted migration or future-proofing strategies, requires a two-step process. First, the projected future environment at the restoration site is extracted from the future climate layers. The extracted data are then applied to current climate layers to identify grid cells that currently match the site’s projected future conditions.

*Extension to regional matching*

We extended the basic Restore and Renew site-matching method for cases where a region, rather than a single site, is the target for restoration. All grid cells within the regional polygon were used to define the observed range for each of the six environmental variables used in the Restore and Renew webtool. Matches were defined as grid cells whose values for all selected variables fell within the corresponding ranges observed within the region.

*Application to Big Scrub* restoration

For the present study, regional environmental matching was performed using two fundamental climate variables: mean annual temperature and mean annual rainfall. Our previous experience developing and applying the Restore and Renew webtool indicates that these two variables effectively define broad environmental matches to guide the selection of collection locations. Limiting the present study to these variables also minimised the constraints imposed by the “curse of dimensionality”.

As noted above, we acknowledge the limitations of using a restricted set of environmental variables to define a broad envelope for selecting matching locations. In particular, soil conditions are recognised as critical for plant species. The Restore and Renew webtool therefore recommends using the basic matched regions as candidate areas, then refining them using other information sources, such as local knowledge of intact native vegetation, known species occurrences, or soil characteristics assessed directly in the field.

In the case of the Big Scrub region, soils are derived primarily from basalt outflows. Thus, broad matches based on mean annual temperature and rainfall can be overlaid with mapped areas of basalt substrate to further guide the selection of sites for propagation material collection. **Fig. S4** illustrates how this approach could be applied by a restoration practitioner.


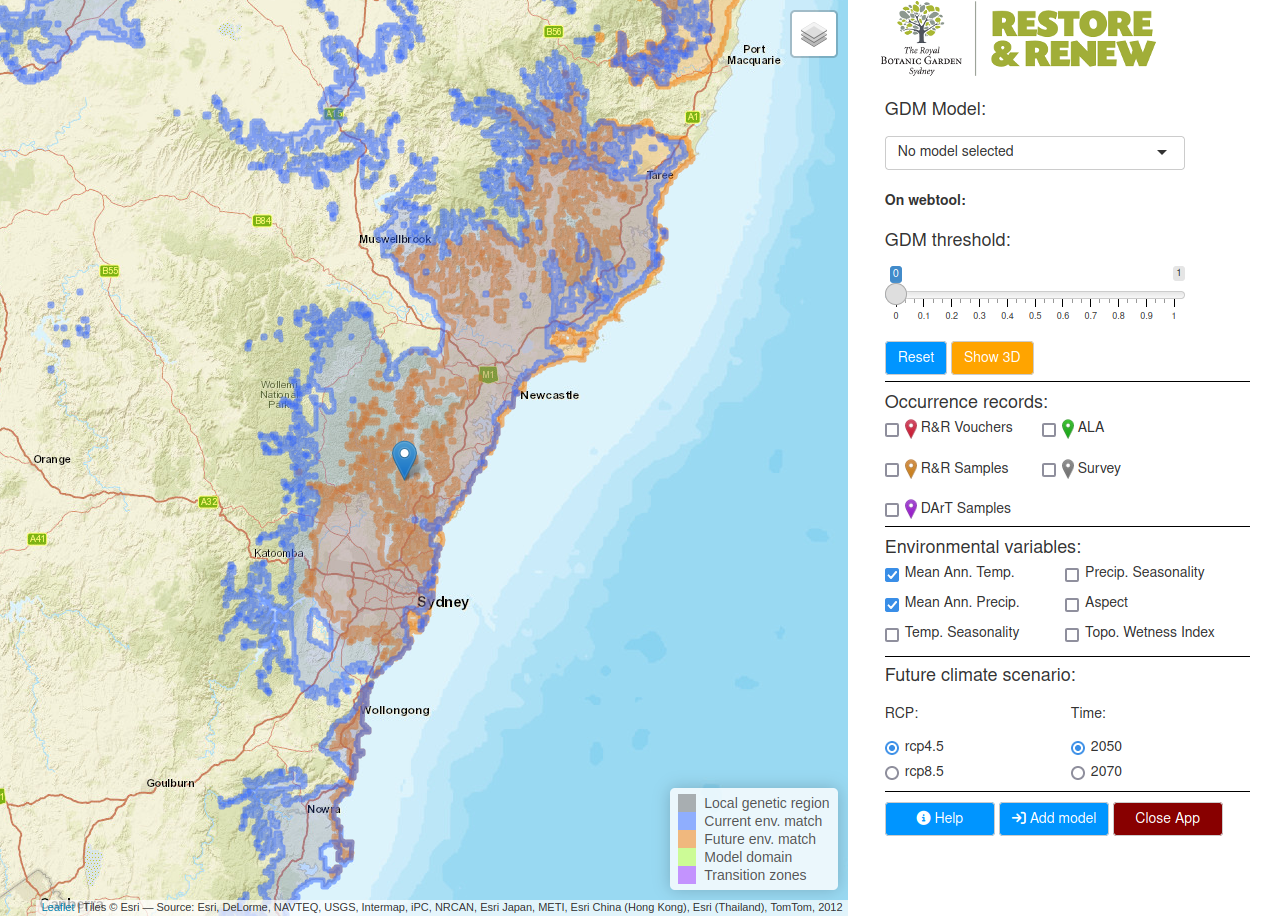


**Fig. S1**: Current and future matched areas for a selected restoration site. Environmental variables selected were Mean Annual Temperature and Mean Annual Rainfall. The application used was a desktop interface to the Restore and Renew Webtool R-package (Rossetto et al 2019).


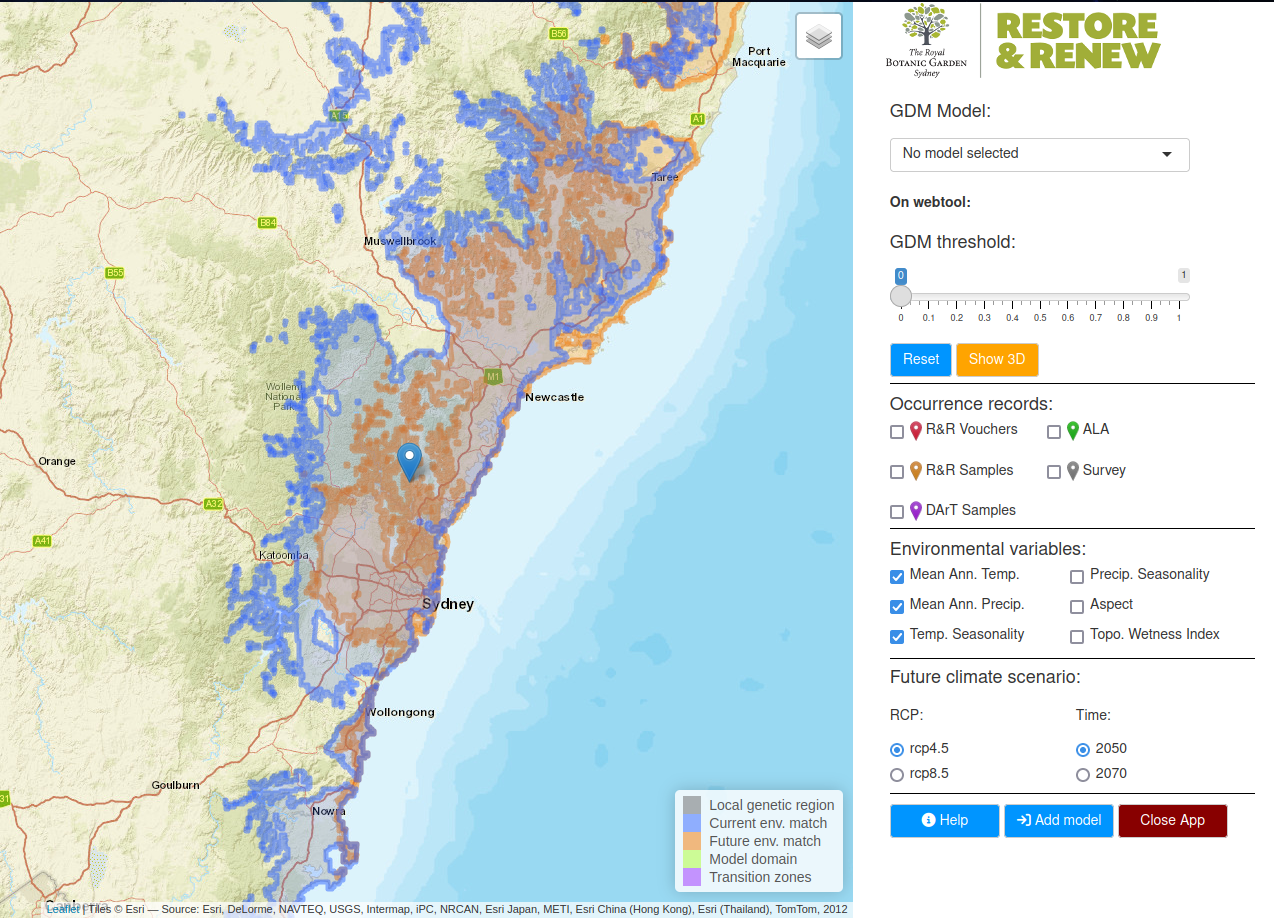


**Fig. S2**: Current and future matched areas for a selected restoration site. Environmental variables selected were Mean Annual Temperature, Mean Annual Rainfall and Temperature Seasonality. The shift in the extent and fragmentation of current and future regions is minor with main changes seen in the western (inland) area.


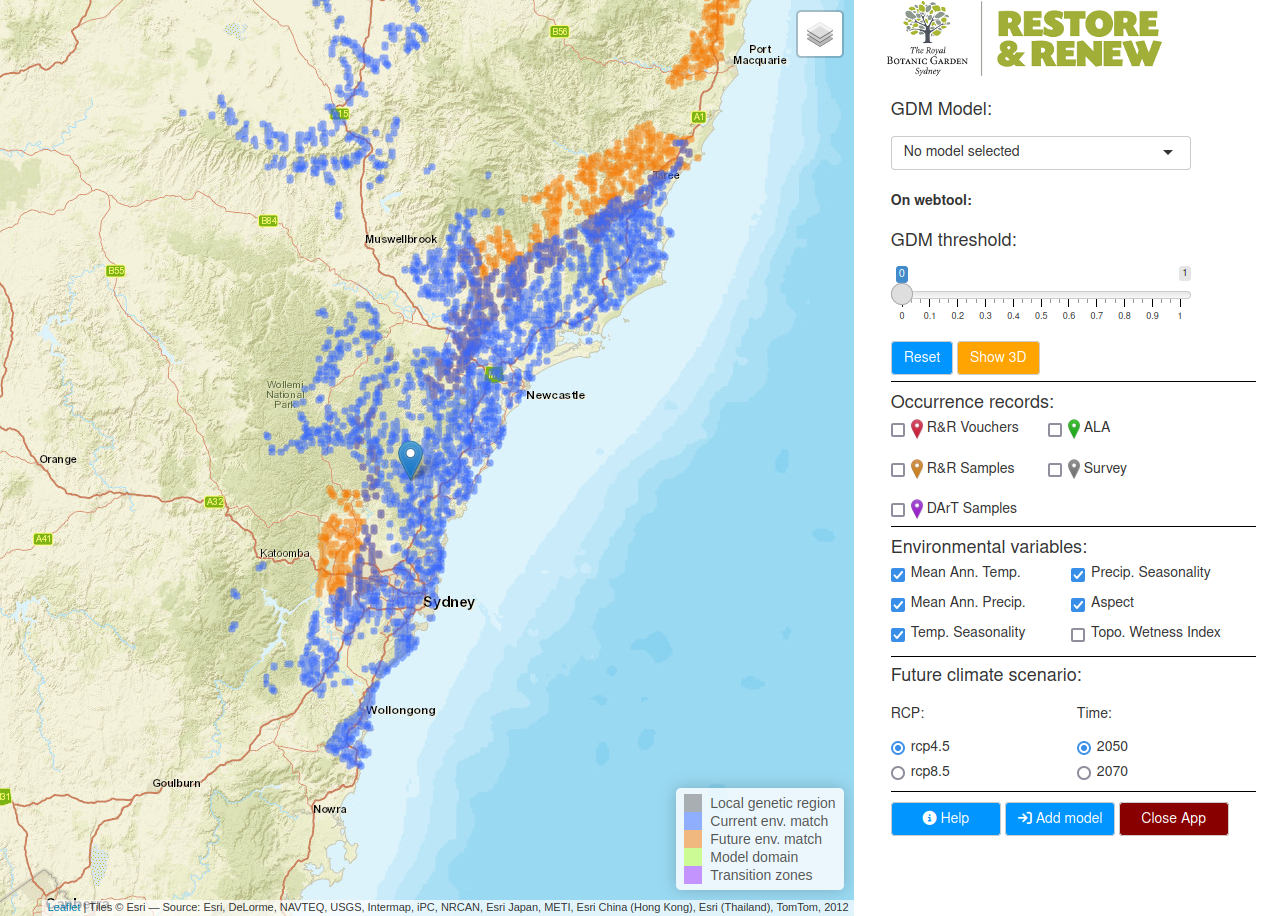


**Fig. S3:** Current and future matched areas for a selected restoration site. Environmental variables selected were Mean Annual Temperature, Mean Annual Rainfall, Temperature Seasonality, Precipitation Seasonality and Aspect. The shift in the extent and fragmentation of current and future regions is extreme relative to figures S1 and S2.


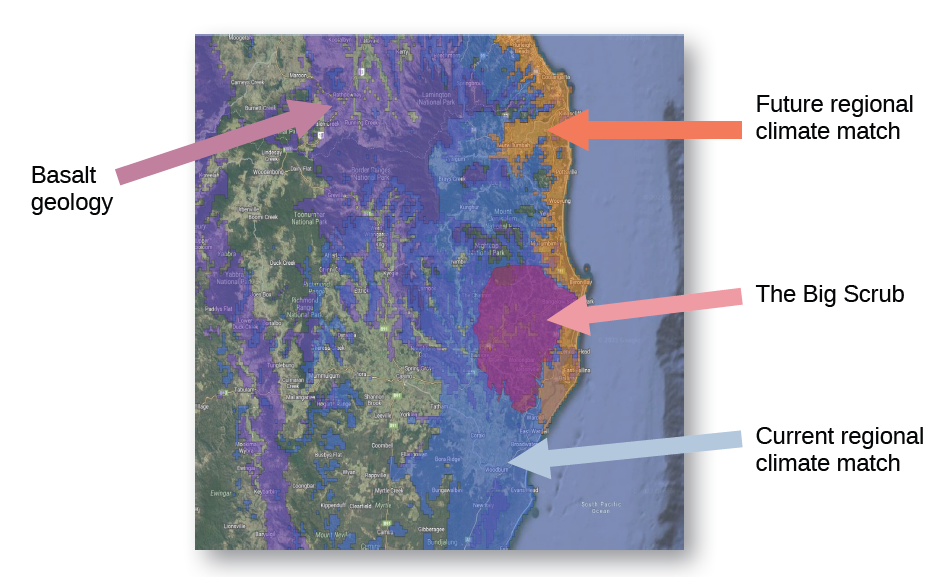


**Fig. S4**: Current and future climate match for the Big Scrub region. Climate variables used were Mean Annual Temperature and Mean Annual Rainfall. Regions with basalt substrate are overlaid to allow restoration practitioners to consider targeting areas with appropriate basalt-derive soils. Future climate data was derived from an ensemble of models produced for the Fifth Assessment Report of the Inter-Governmental Panel on Climate Change (IPCC). A severe climate change scenario (RCP8.5) and climate averages for a 30-year future time period centred on 2070 were used in this example.

**Appendix 2: Additional analyses supporting the identification of Genetic Neighbourhoods (GNs) and zones of admixture**


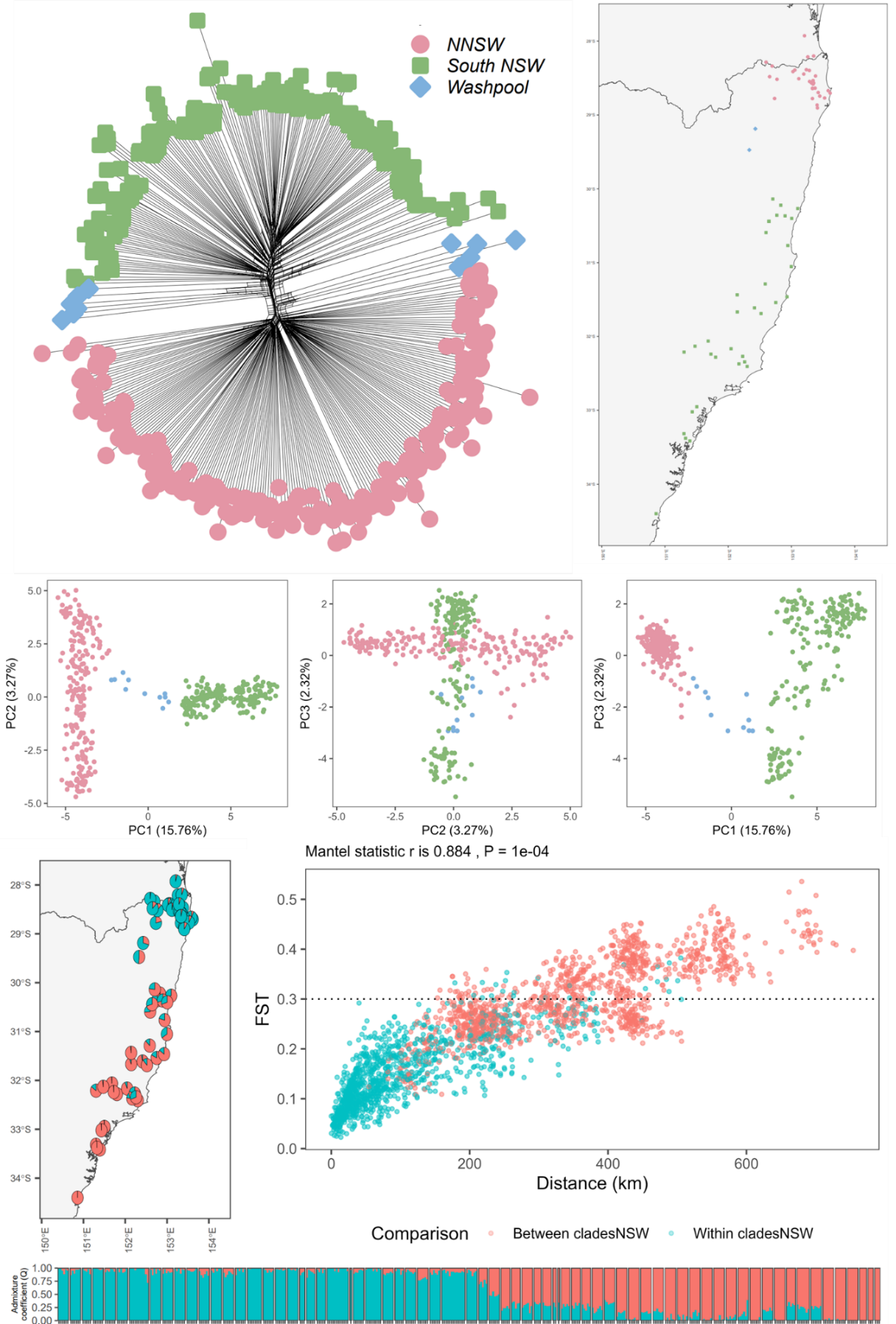


**Fig. S5**: Population genetic analyses (splitstree network, principal component analysis, isolation by distance-F_ST_, and LEA sNMF plots) of *Neolitsea dealbata* clades found within the state of New South Wales (NSW), highlighting the admixture zone between northern and southern GNs around the geographically intermediate Washpool region.


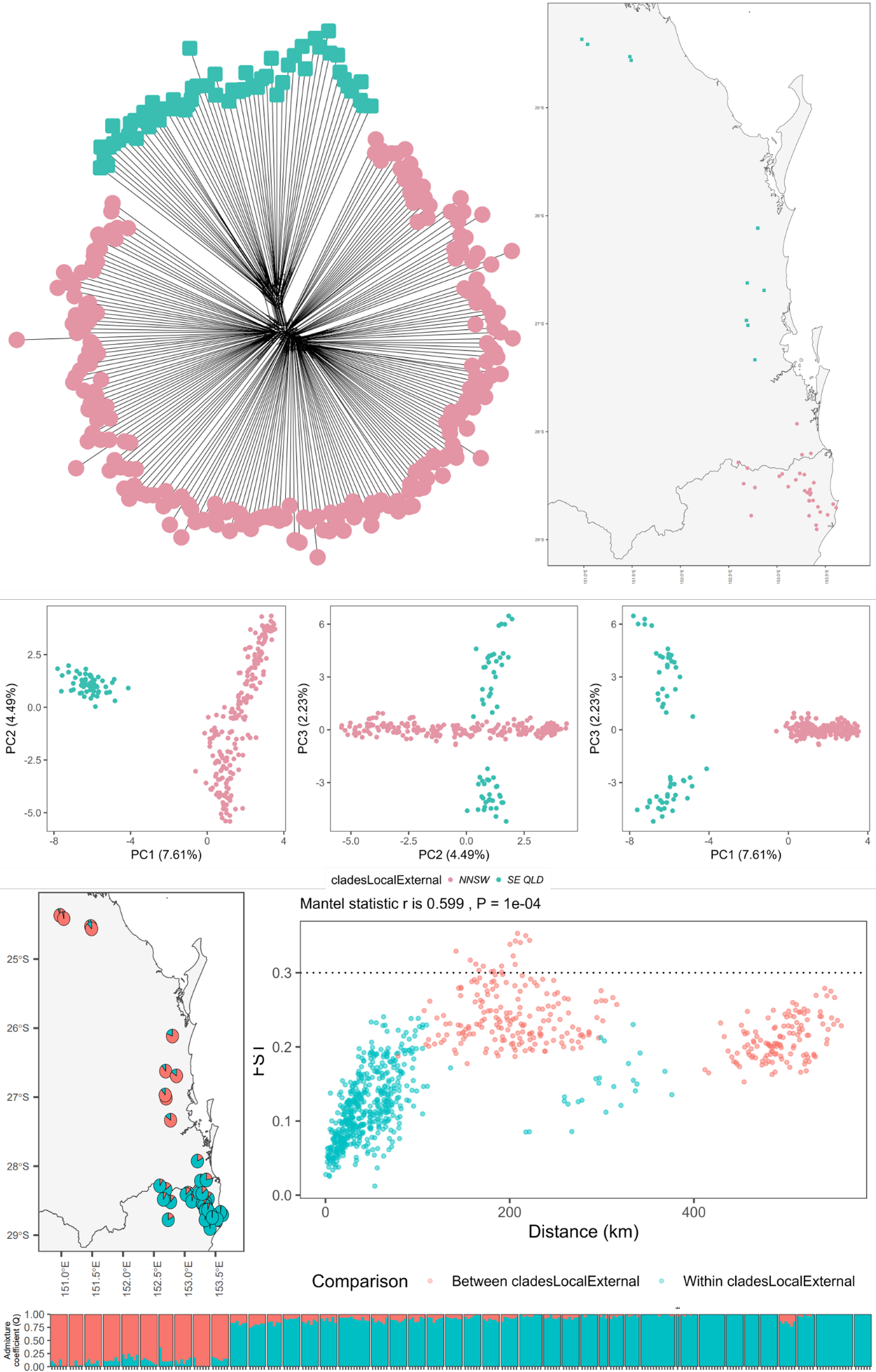


**Fig. S6:** Population genetic analyses (splitstree network, principal component analysis, isolation by distance-F_ST_, and LEA sNMF plots) of local and external genetic neighbourhood (GN) used in this study, indicating strong genetic structuring between GNs while representing FST values >0.5.

**Appendix 3: Frequency of sites and individuals across the *local* and *external GN* for both unconstrained and constrained datasets**

**Table S1:** Provenancing scenarios A–C incorporated in this study, along with related terminology from the literature. Applied examples based on the local and external genetic neighbourhoods outlined in **Fig. 1** are included.

| Applied Genetic Neighbourhood Provenancing Scenarios | Current terms used in the literature | References | Applied GN Example |
| --- | --- | --- | --- |
| Scenario A –  Local Provenancing  (*Local GN*) | Local provenancing  Admixture provenancing (within *local GN*)  Composite provenancing (within *local GN*)  Strict local provenance  Relaxed local provenance  Regional admixture provenancing | (Breed et al. 2018; Broadhurst et al. 2008; Bucharova et al. 2019; Harrison et al. 2021; Prober et al. 2015) | 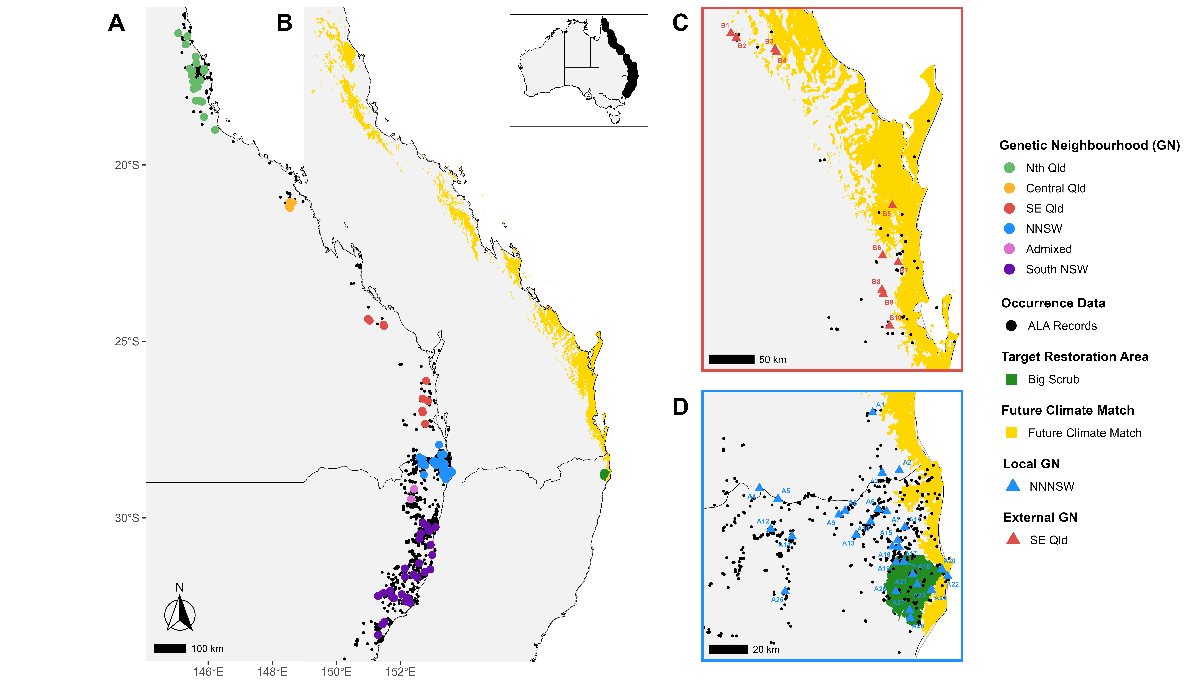  *Local GN* |
| Scenario B –  Predictive Provenancing  (*External GN*) | Predictive provenancing | (Crowe and Parker 2008; Prober et al. 2015; Sgrò et al. 2011) | 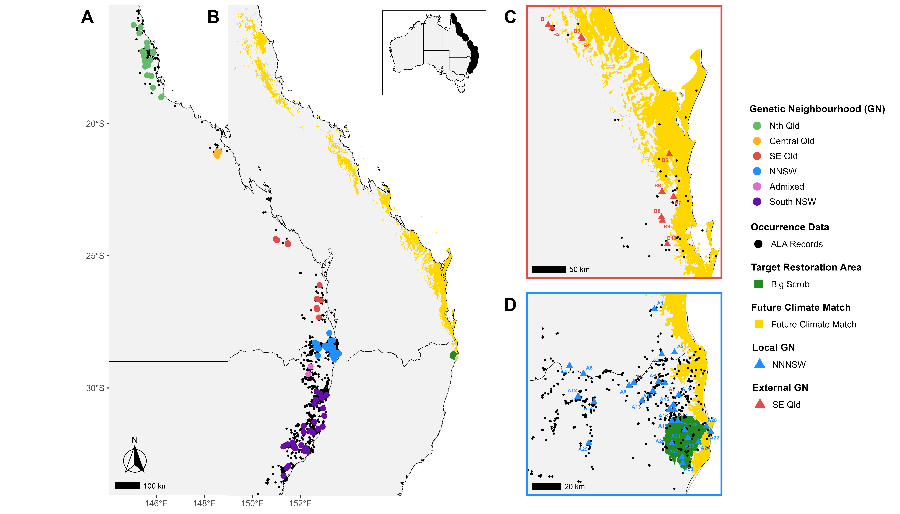  *External GN* |
| Scenario C –  Climate-adjusted Provenancing  (*Local* and *External GN*) | Climate-adjusted provenancing  Admixture provenancing  Composite provenancing | (Broadhurst et al. 2008; Harrison et al. 2021; Prober et al. 2015) | 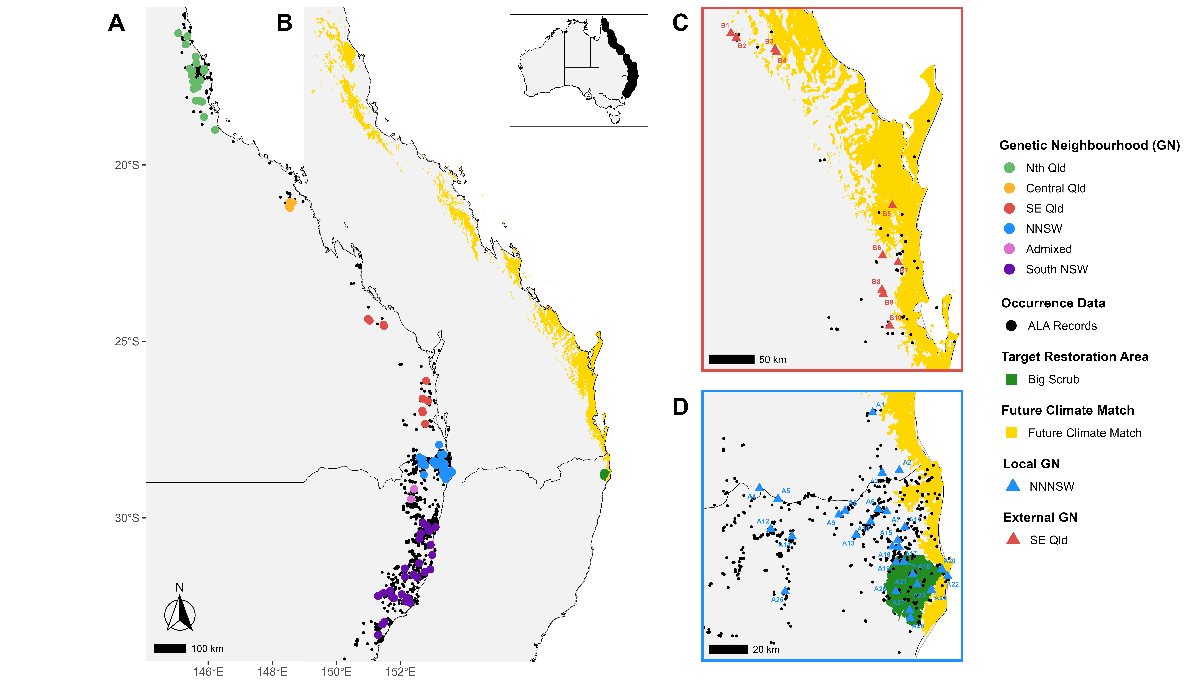  +  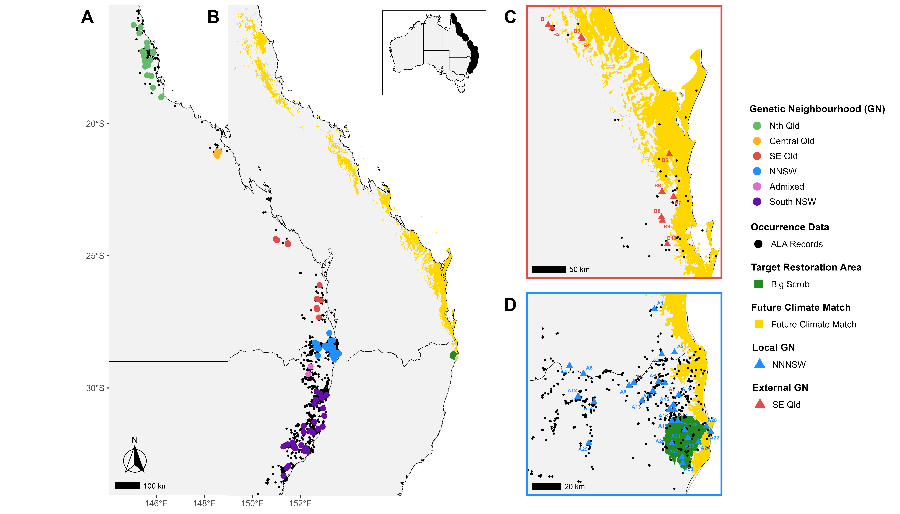  *Local GN* + *External GN* |

**Table S2:** Total number of sites and individuals per site represented for the local and *external GN*s used throughout this study. Sites/individual which were forced inclusion or exclusion in the constrained sampling scenarios used in this study are also indicated.

| Genetic Neighbourhood | Site | Individuals | Constrained Scenario |
| --- | --- | --- | --- |
| Local | A1 | 6 | Excluded |
| Local | A2 | 6 | Excluded |
| Local | A3 | 5 | Excluded |
| Local | A4 | 6 | Excluded |
| Local | A5 | 6 | Excluded |
| Local | A6 | 6 | Excluded |
| Local | A7 | 6 | Excluded |
| Local | A8 | 5 | Excluded |
| Local | A9 | 6 | Excluded |
| Local | A10 | 6 | Excluded |
| Local | A11 | 6 |  |
| Local | A12 | 6 | Excluded |
| Local | A13 | 6 | Excluded |
| Local | A14 | 6 | Excluded |
| Local | A15 | 6 |  |
| Local | A16 | 6 |  |
| Local | A17 | 6 |  |
| Local | A18 | 5 |  |
| Local | A19 | 6 |  |
| Local | A20 | 6 |  |
| Local | A21 | 5 |  |
| Local | A22 | 6 |  |
| Local | A23 | 5 | Included |
| Local | A24 | 6 | Included |
| Local | A25 | 6 | Excluded |
| Local | A26 | 6 |  |
| Local | A27 | 6 |  |
| Local | A28 | 6 | Included |
| External | B1 | 6 | Excluded |
| External | B2 | 5 | Excluded |
| External | B3 | 6 |  |
| External | B4 | 6 |  |
| External | B5 | 6 |  |
| External | B6 | 6 |  |
| External | B7 | 5 |  |
| External | B8 | 6 | Included |
| External | B9 | 6 |  |
| External | B10 | 6 | Included |

**Appendix 4. Detailed implementation and outputs of *psfs*-based optimisation**

*1. Calculation of the pSFS diversity measure*

*1.1 Grouping by missingness*

To ensure accurate SFS estimation under variable missing data, loci were grouped based on the number of individuals with missing genotypes. Within each group, the minor allele frequency distribution (site frequency spectrum; SFS) was calculated.

For individual-based optimisation, minor allele counts were derived per individual using the *gt_to_minor_alleles* function, whereas for site-based optimisation, allele counts were first aggregated by site before calculating the SFS using the *gt_to_pop_minor_allele_counts* function (*sfsCalcs* package: <https://github.com/recer-rbgsydney/sfsCalcs>).

*1.2 Down-projection*

Each group-specific SFS was down-projected to a fixed number (m) following Marth  *et al.* (2004). This projection standardises sample sizes across loci which helps reduce bias due to missing data. For all *psfs* optimisations, *m* equalled the total number of target individuals wanting to be optimised.

*1.3 Final* psfs *metric*

Each down-projected SFS was summed across loci to generate a composite SFS, and the proportion of non-fixed SNPs (i.e., loci with both alleles present) was used as the measure of allelic diversity, i.e., *psfs* = number of non-fixed loci/ total number of loci. This measure was computed using the *psfs_diversity* function in the *OptGenMix* package (<https://github.com/recer-rbgsydney/OptGenMix>) which calculates the projected SFS using the *project_SFS_from_genotypes* and *project_SFS_from_MAC* functions from the accompanying *sfsCalcs* package (<https://github.com/recer-rbgsydney/sfsCalcs>) depending if the psfs is calculated for individual or site combinations respectively. Visual examples of *psfs* calculations for individuals and sites are shown in **Fig. S7** and S8, respectively.

2. Simulated annealing optimisation

*2.1 Algorithm summary*

The simulated annealing algorithm searches for the optimal combination of samples or sites that maximises a target objective function (here, the *psfs* metric). It iteratively proposes alternative combinations, accepts improved solutions deterministically, and accepts less optimal ones with a probability governed by a temperature parameter, allowing the search to escape local maxima and approach a global optimum.

*2.2. Constrained optimisations*

Forced inclusion or exclusion of samples (Decision 2) was implemented via the “initial_weights”, “weights_min”, and “weights_max” parameters in the *optimize_single_objective* function. For any forced inclusion, initial_weights = 1 and weights_min = 1. For any forced exclusion, initial_weights = 0`and weights_max = 0. These parameters fixed the inclusion or exclusion status of specific individuals or sites during the optimisation search, allowing complimentary combinations to be optimised with freely available samples remaining.

*2.3 Implementation and parameter settings of* OptGenMix

Apart from applied constraints, the optimisation was performed using the *optimize_single_objective* function from the *OptGenMix* package (sfs branch) with the the measure argument was set to "psfs" to specify the *psfs* diversity measure.

| Parameter | Description | Value |
| --- | --- | --- |
| max_steps | Number of iterations in annealing chain | 10,000 |
| max_t | Maximum temperature (controls initial randomness) | 0.5 (individual), 0.001 (site) |
| min_t | Minimum temperature (convergence threshold) | 0 |
| m | Target number of chromosomes for down-projection | = number of individuals or sites being optimised |
| measure | Optimisation target function | "psfs" |

Convergence was confirmed by plotting the optimisation temperature profile to ensure gradual decline and stabilisation near the global optimum.


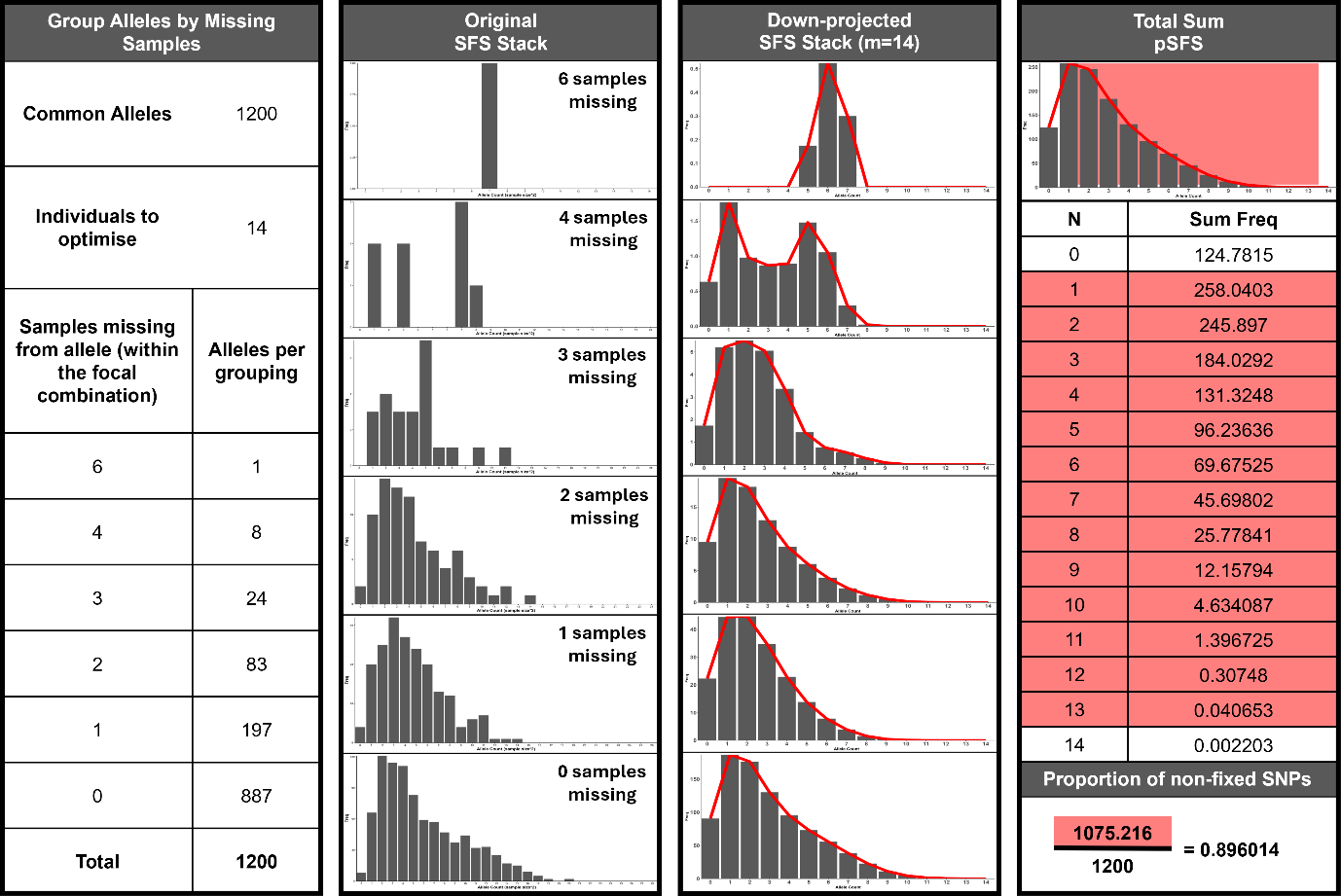


**Fig. S7:** Calculating the *psfs* measure used to optimise individual-based sampling combinations in this study. The figure details the process of a down-projected site frequency spectrum (SFS) stack of 14 sampled individuals. For each SFS, the x-axis represents allele counts, and the y-axis indicates the frequency. An example using site-based combinations is presented in **Fig. S8**.


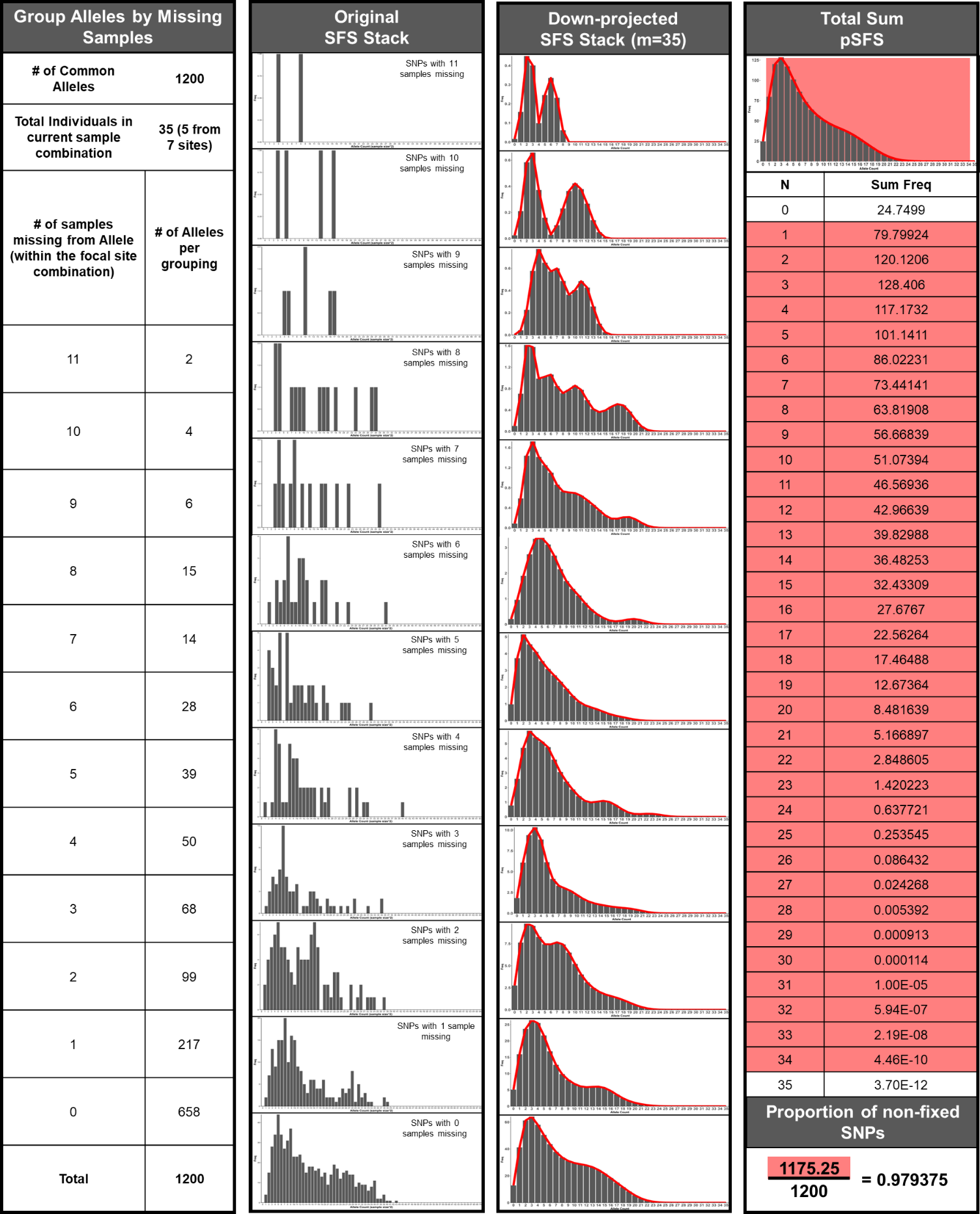


***Fig. S8:*** *Calculating the psfs measure used to optimise site-based sampling combinations in this study. The figure details the process of a down-projected site frequency spectrum (SFS) stack of 5 individuals per site across 7 sites, representing a total of 35 sampled individuals. An example using individual-based combinations is presented in* ***Fig. S7****.*

*3. Associated outputs from psfs optimisation*


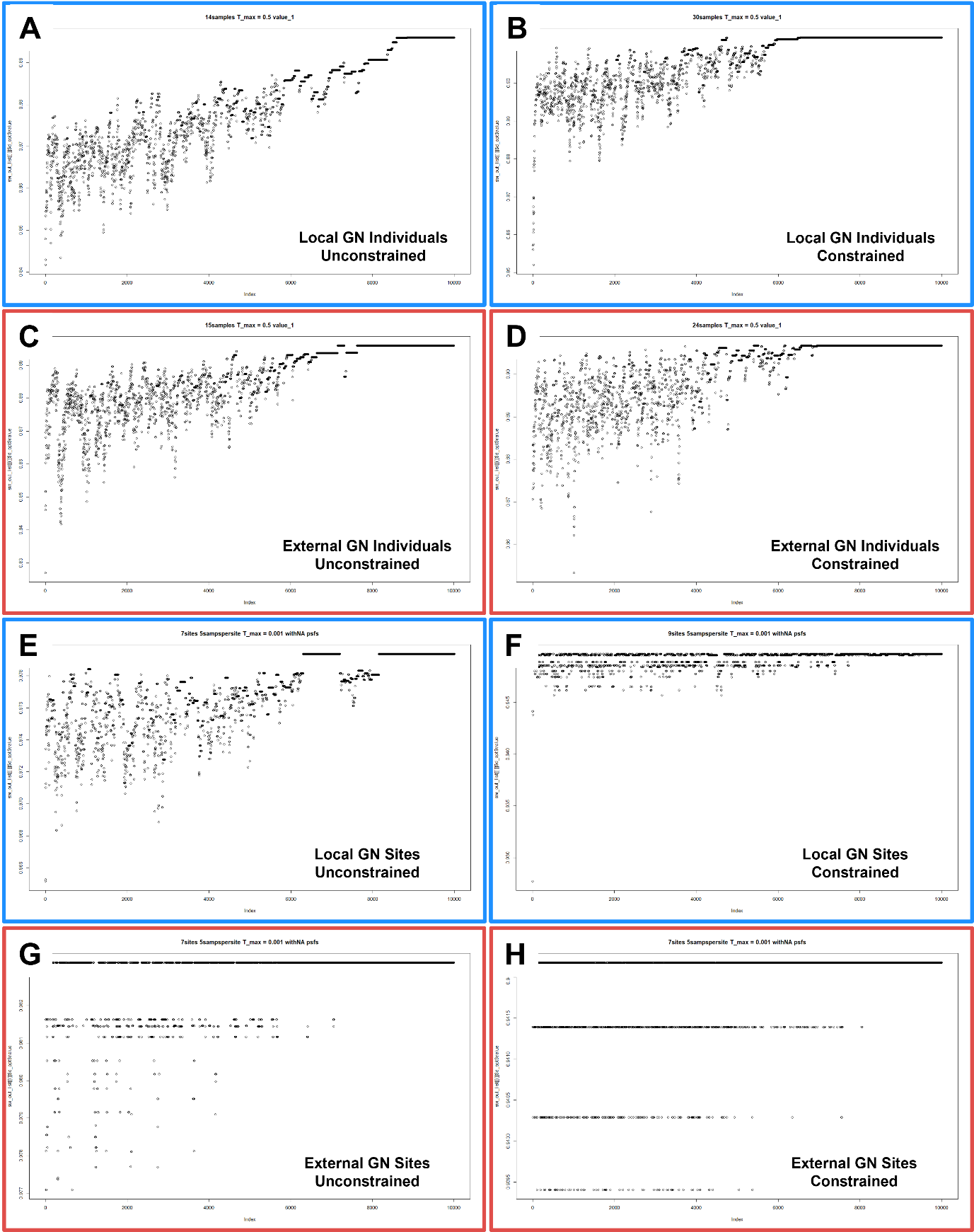


**Fig. S9**: Temperature plots of unconstrained and constrained simulated-annealing for both individual-based (1-4) and site-based (5-8) optimisation across scenario A - *local GN* (1-2 & 5-6) and scenario B - *external GN* (3-4 & 7-8). Max steps for each run = 10000. Max temp for individual-based optimisation=0.5, while max temp for site-based optimisation=0.001.


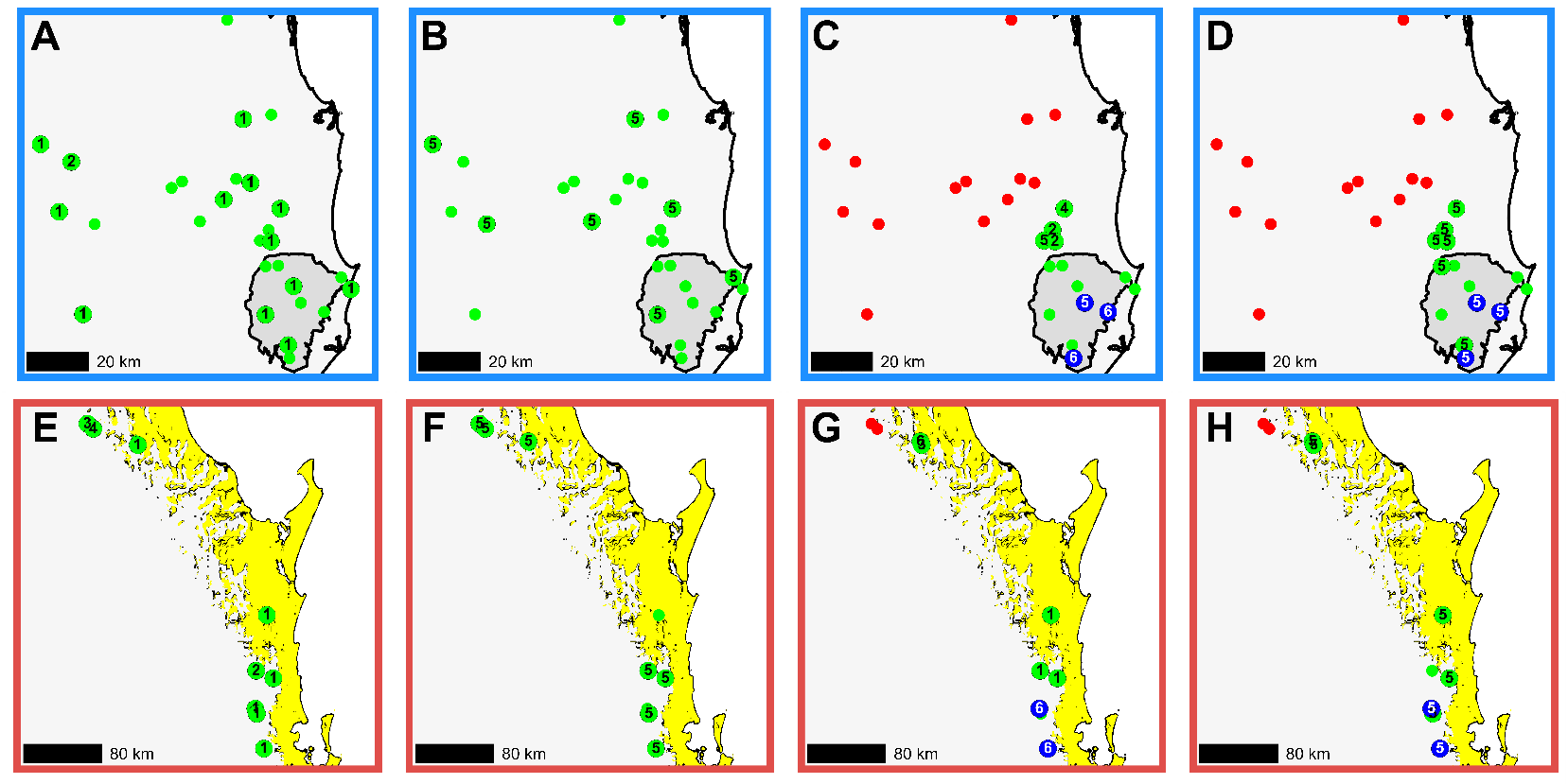


**Fig. S10:** Map showing the distribution of optimised sites and number of samples per site identified for both unconstrained (A-B, E-F) and constrained (C-D, G-H) sampling strategies for restoration scenario A (*local GN*; A-D) and B (*external GN*; E-H). Green points represent freely available (unconstrained) sites. The dark grey region represents the target restoration area (Big Scrub). The yellow highlighted area represents the regional future climate match of the target restoration area. Red circles represent sites that were excluded from the constrained scenario. Blue circles represent sites with forced inclusion from implemented constraints.


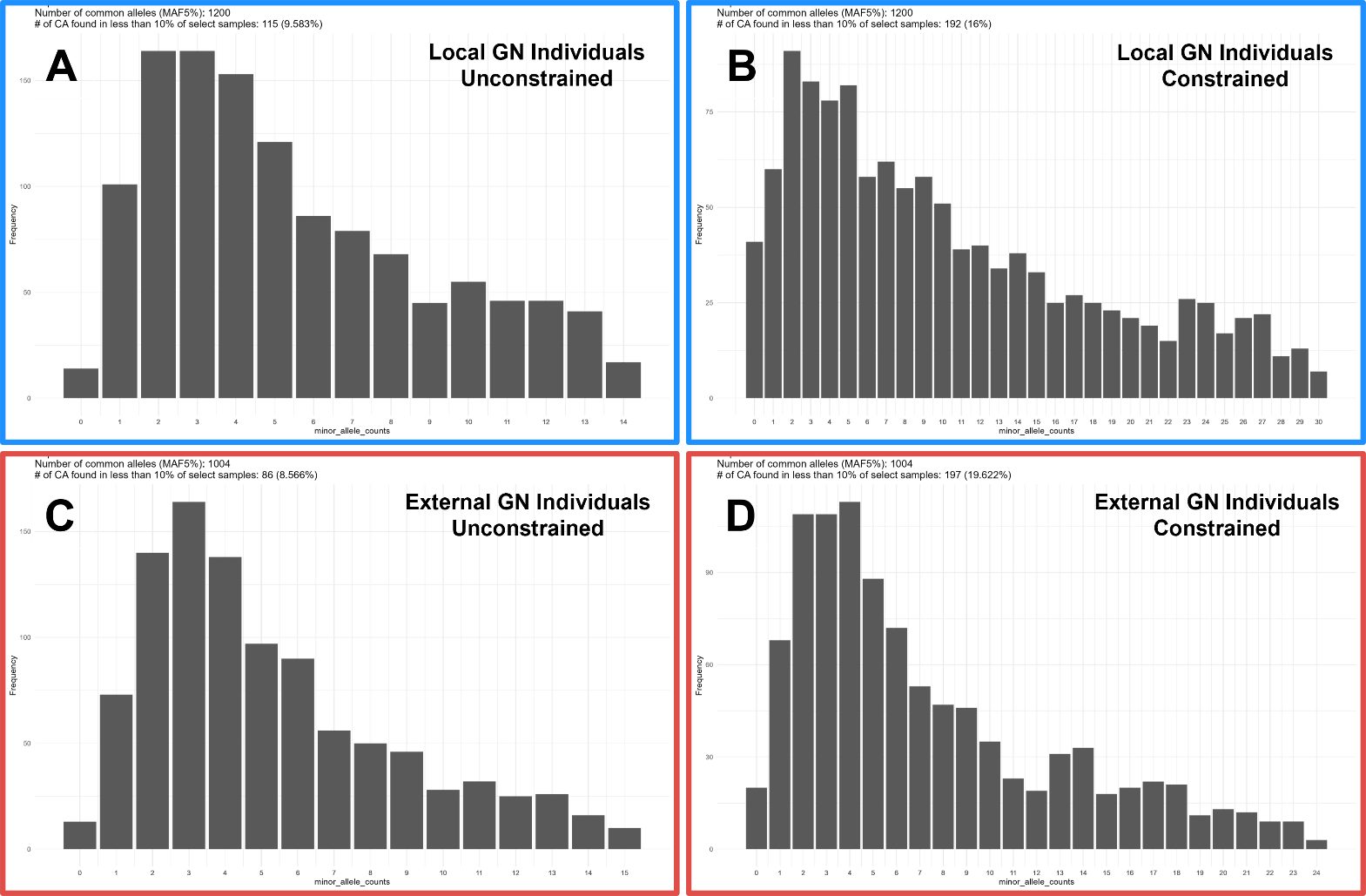


**Fig. S11:** Frequency of common alleles observed within optimised individual samples for both the local (A-B) and external (C-D) GN presented for both unconstrained (A & C) and constrained (B & D) outcomes. The total number of common alleles is outlined in the title for each figure.

**Appendix 5: Detailed Methods and Outputs from Validation Testing of Sampling Outcomes**

The following section outlines the detailed methods applied to three validation tests designed to evaluate sampling outcomes for provenancing material within the *local GN* (Scenario A).

*Test 1: Removal of samples from the final optimised combinations*

To evaluate the potential impact of propagule mortality (e.g., failure of individuals to survive propagation or transplantation into the final SPA), we tested the effect of removing every possible combination of 1–5 individuals from the unconstrained optimised dataset for provenancing Scenario A. This was applied to both the minimised-site and minimised-individual approaches described under Decision 1.

For each removal scenario, we calculated the resulting proportion of common alleles retained in the remaining subset. This test assessed whether optimised SPA plantings can still represent at least 90% of common alleles despite moderate levels of mortality, or whether replacement of each lost individual is required to maintain genetic representativeness.

*Test 2: Impact of site removal on random sampling within the* local GN *dataset*

This test evaluated whether the sampling density and spatial distribution of sites within the *local GN* dataset influenced the outcomes of the SPA design process. Specifically, it assessed the robustness of the randomisation methods used to determine the minimum number of samples required to represent common alleles when site composition is altered.

To test this, we reanalysed the unconstrained outcomes for Scenario A using a reduced set of sites. Instead of the full set of 28 sites, we applied two subsampling approaches that halved the number of sites: (A) geographically representative sites across the *local GN*, and (B) geographically biased sites restricted to one region of the *local GN*. The list of sites retained for each subsampling approach is provided in Tables S3-S4 (**Appendix 5**).

We then compared these randomised results with the original outputs for both the site- and individual-minimisation approaches (Decision 1) to evaluate whether changes in site composition affected the number of samples required to capture at least 90% of common alleles.

*Test 3: Alternative sampling guidelines for fixed (optimised) combinations*

In real-world restoration efforts, some individuals selected through optimisation may become unavailable for sampling—often only discovered during site visits. Common reasons include mortality, missing plant tags, or lack of seed set (if collecting seed as the propagation method), which are frequently encountered during in situ sampling (R. Dimon, pers. obs.).

Although datasets can be reanalysed to exclude such individuals, this may be impractical once significant time and resources have been invested in fieldwork. To simulate this scenario, we assessed the impact of replacing unavailable optimised individuals from Scenario A’s unconstrained individual- and site-based optimisations. Specifically, we randomly substituted these “unavailable” individuals with alternative individuals from the same site, assuming that intra-site genetic similarity allows for functional replacement.

We then compared the proportion of common alleles captured in these substituted combinations with the original optimised outcomes to determine whether the >90% representation threshold for common alleles in the *local GN* could still be maintained.

**Table S3:** Sites removed to test resilience of optimised outcomes against effects of sampling density. The remaining sites once halved represented an even geographical spread across the *local GN*, or a biased geographical area across the *local GN*.

| Site | Individuals | Half the number of sites (even geographical spread) | Half the number of sites (biased geographical spread) |
| --- | --- | --- | --- |
| A1 | 6 | ✔ |  |
| A2 | 6 | ✔ |  |
| A3 | 5 | ✔ |  |
| A4 | 6 |  |  |
| A5 | 6 |  |  |
| A6 | 6 |  |  |
| A7 | 6 |  |  |
| A8 | 5 | ✔ |  |
| A9 | 6 |  |  |
| A10 | 6 |  |  |
| A11 | 6 |  | ✔ |
| A12 | 6 |  |  |
| A13 | 6 |  |  |
| A14 | 6 | ✔ |  |
| A15 | 6 | ✔ | ✔ |
| A16 | 6 |  | ✔ |
| A17 | 6 |  | ✔ |
| A18 | 5 | ✔ | ✔ |
| A19 | 6 | ✔ | ✔ |
| A20 | 6 | ✔ | ✔ |
| A21 | 5 | ✔ | ✔ |
| A22 | 6 |  | ✔ |
| A23 | 5 | ✔ | ✔ |
| A24 | 6 |  | ✔ |
| A25 | 6 |  |  |
| A26 | 6 | ✔ | ✔ |
| A27 | 6 | ✔ | ✔ |
| A28 | 6 | ✔ | ✔ |

**Table S4:** Comparing sampling outcomes of the removal of sites from the dataset; original sampling number vs half the number of sites while maintaining geographical representation vs half the number of sites with biased geographical representation.

| Unconstrained local scenario | All sites in *local GN* | Half the number of sites (even geographical spread) | Half the number of sites (biased geographical spread) |
| --- | --- | --- | --- |
| Map of sites selected (Green circles; **Table S2**) | 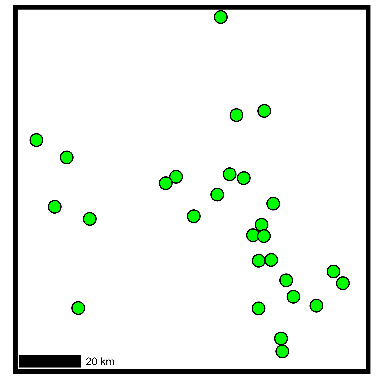 | 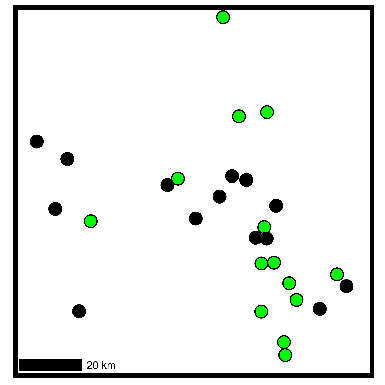 | 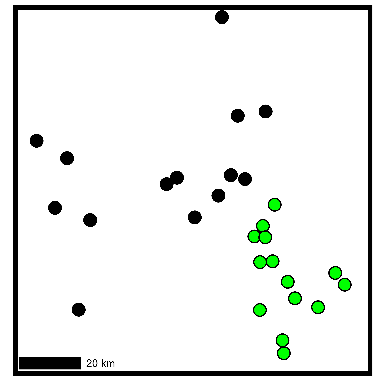 |
| # sites in dataset | 28 | 14 | 14 |
| Total SNPs in dataset | 6132 | 4160 | 3879 |
| # Common Alleles | 1200 | 1189 | 1040 |
| # Indiv. to capture 90% Common alleles | 14 | 14 | 12 |
| # sites to capture 90% Common alleles | 35 (5 Indiv. from 7 sites) | 35 (5 Indiv. from 7 sites) | 20 (5 Indiv. from 4 sites) |


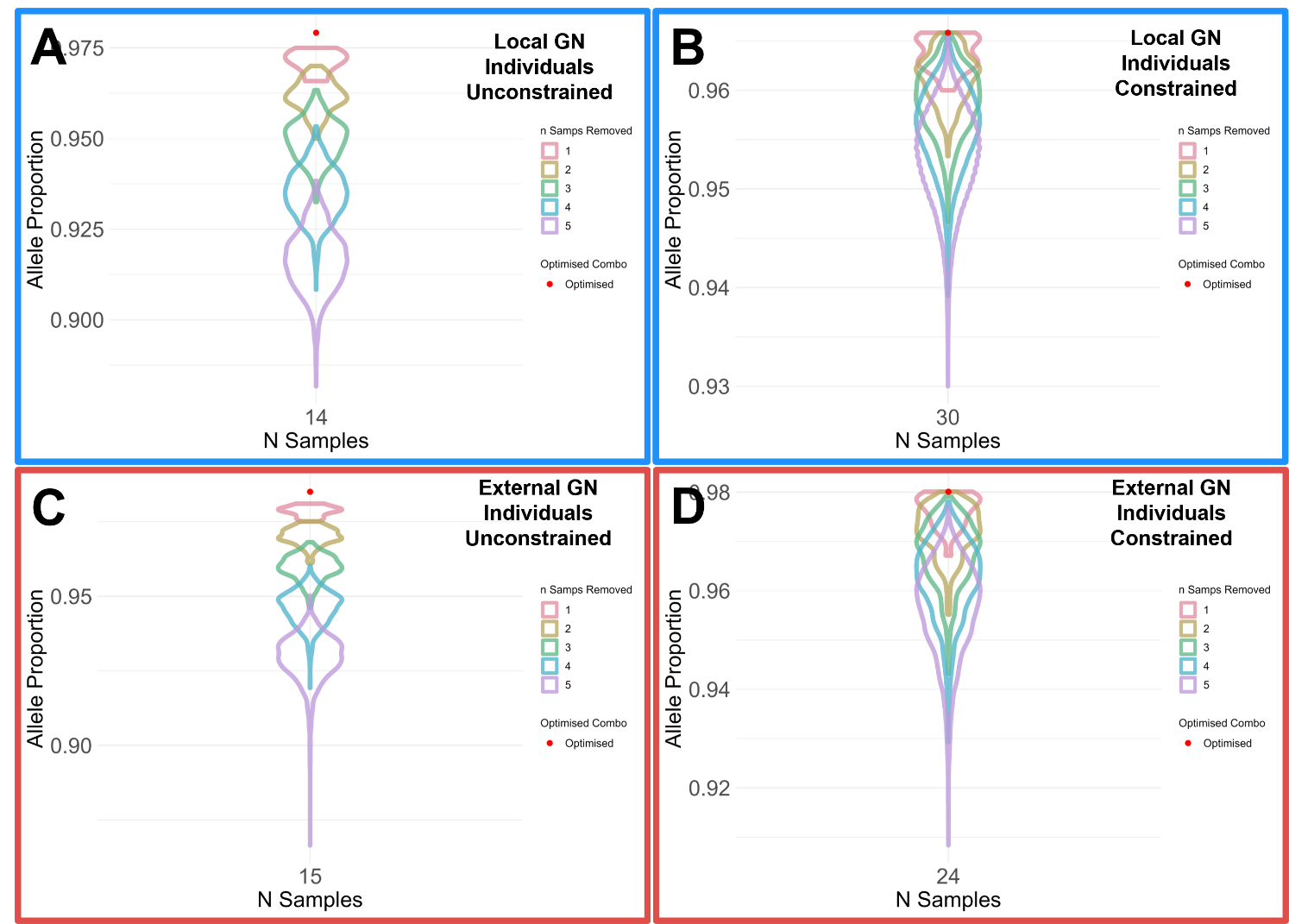


**Fig. S12:** Violin plots showing the range of common allele proportion remaining when 1-5 individuals are removed from optimised sampling combinations. Ranges are presented for the local (A-B) and external (C-D) GN using individual-based optimisation for unconstrained (A & C) and constrained (B & D) combinations. the x axis shows the starting number of optimised individuals, for which common allele proportion is presented as a red dot.


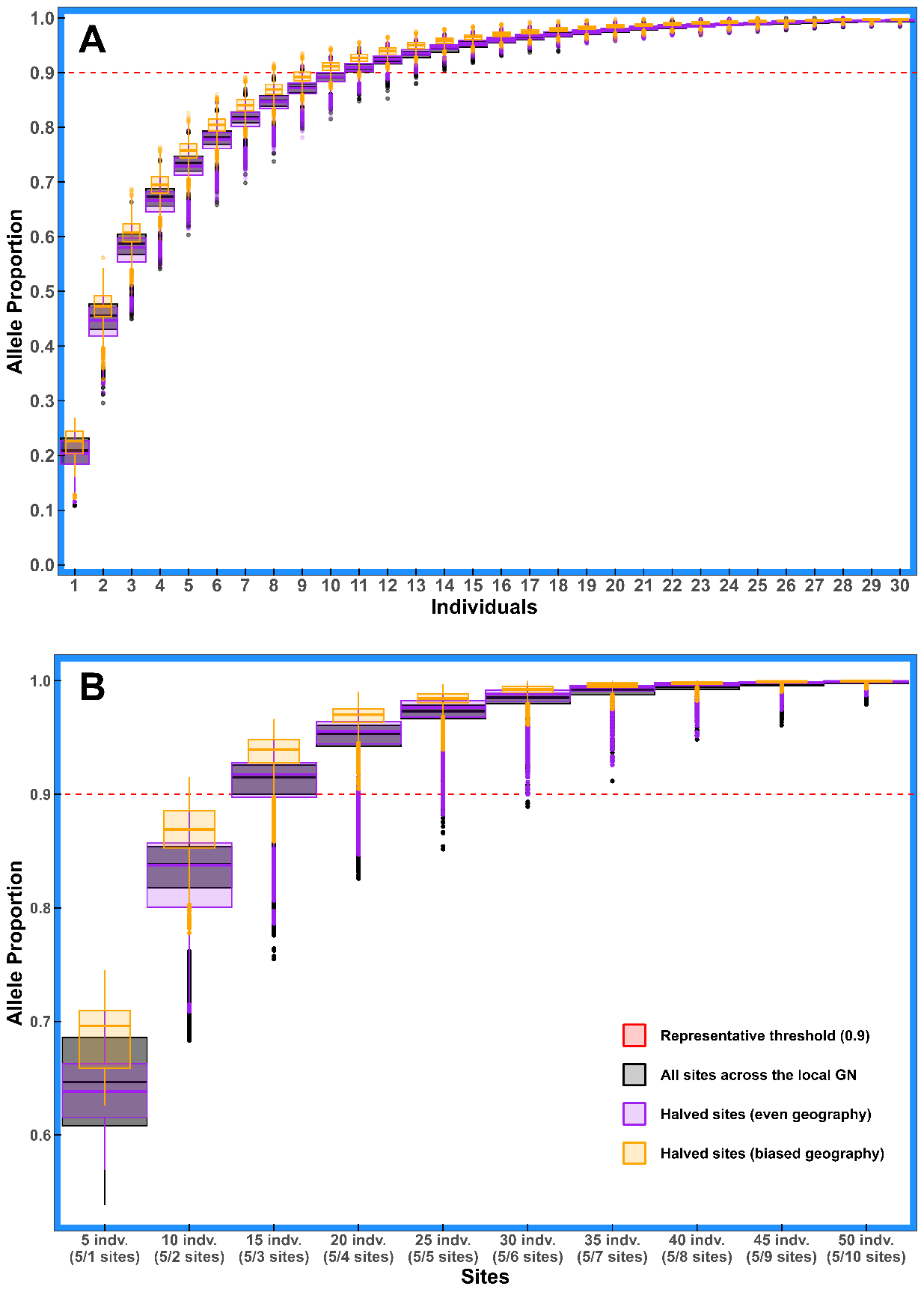


**Fig. S13:** Boxplots of individual (A) and site-based (B) common allele capture comparing sampling outcomes when removing half the number of sites from the *local GN* and reanalysing the subsequent dataset; Common allele capture for the original dataset (24 sites) in grey is similar in allele capture when the number of sites is halved, but geographical representation across the *local GN* is maintained (purple). In comparison, common allele capture is over-represented for the *local GN* when half the number of sites is biased to a geographic subset with the *local GN* (orange).


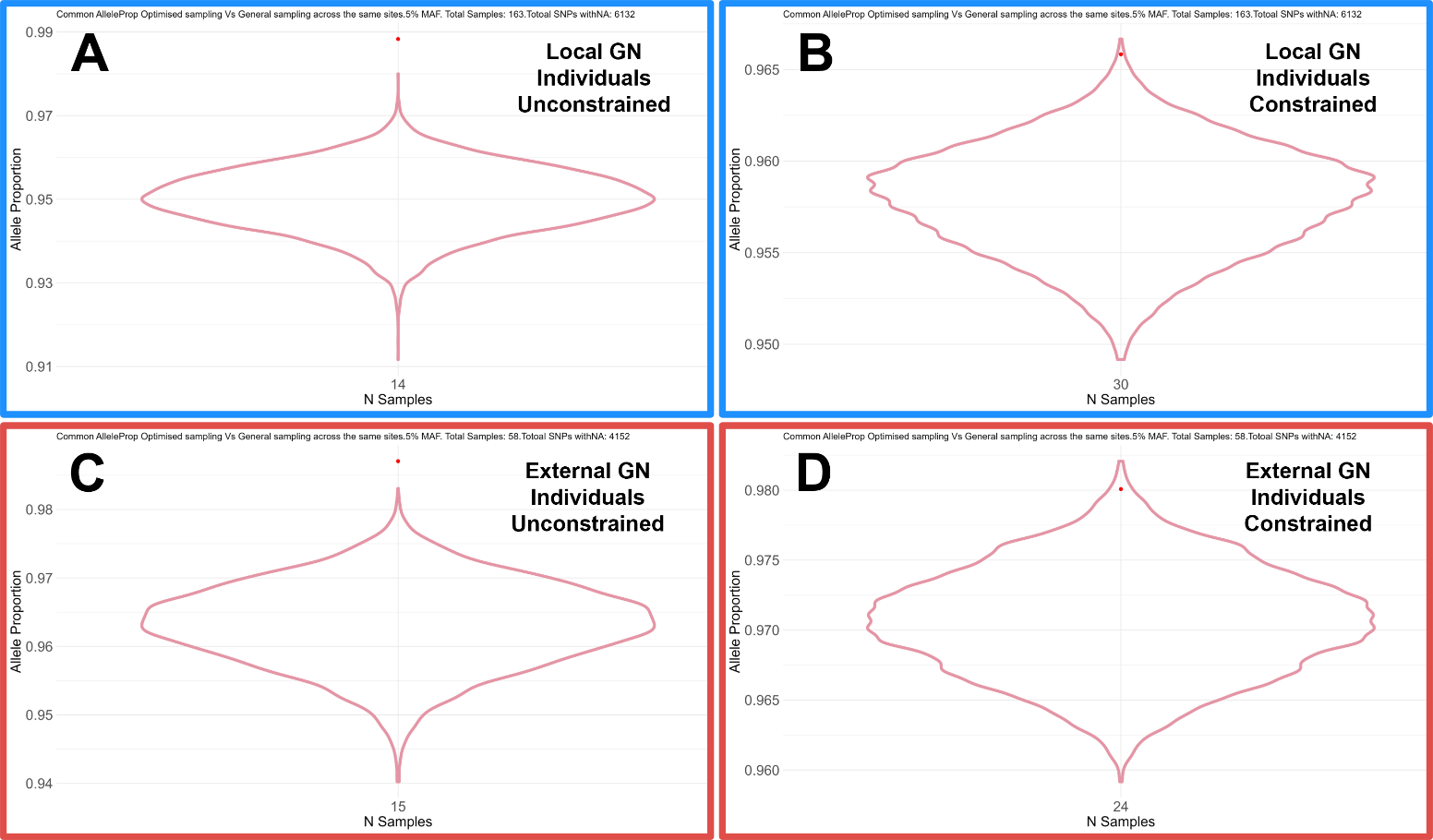


**Fig. S14:** Common alleles represented when optimised individual combinations (red dot) are replaced with alternative individuals found at the same site (violin plots) for both the local (A-B) and external (C-D) GN, across both unconstrained (A & C) and constrained (B & D) combinations using the individual-based approach.

**References**

Breed, Martin F, Peter A Harrison, Armin Bischoff, et al. 2018. “Priority Actions to Improve Provenance Decision-Making.” *BioScience* 68 (7): 510–16.

Broadhurst, Linda M., Andrew Lowe, David J Coates, et al. 2008. “Seed Supply for Broadscale Restoration: Maximizing Evolutionary Potential.” *Evolutionary Applications* 1 (4): 587–97.

Broadmeadow, MSJ, D Ray, and CJA Samuel. 2005. “Climate Change and the Future for Broadleaved Tree Species in Britain.” *Forestry* 78 (2): 145–61.

Bucharova, Anna, Oliver Bossdorf, Norbert Hölzel, Johannes Kollmann, Rüdiger Prasse, and Walter Durka. 2019. “Mix and Match: Regional Admixture Provenancing Strikes a Balance among Different Seed-Sourcing Strategies for Ecological Restoration.” *Conservation Genetics* 20 (1): 7–17. https://doi.org/10.1007/s10592-018-1067-6.

Crowe, Kevin A, and William H Parker. 2008. “Using Portfolio Theory to Guide Reforestation and Restoration under Climate Change Scenarios.” *Climatic Change* 89 (3): 355–70.

Harrison, Peter A, Martin F Breed, Linda M. Broadhurst, et al. 2021. “Florabank Guidelines Module 5: Seed Sourcing.” In *Florabank Guidelines: Best Practice Guidelines for Native Seed Collection and Use*. Florabank Consortium.

Harrison, Peter A., René E. Vaillancourt, Rebecca M. B. Harris, and Brad M. Potts. 2017. “Integrating Climate Change and Habitat Fragmentation to Identify Candidate Seed Sources for Ecological Restoration.” *Restoration Ecology* 25 (4): 524–31. https://doi.org/10.1111/rec.12488.

Marth, Gabor T, Eva Czabarka, Janos Murvai, and Stephen T Sherry. 2004. “The Allele Frequency Spectrum in Genome-Wide Human Variation Data Reveals Signals of Differential Demographic History in Three Large World Populations.” *Genetics* 166 (1): 351–72. https://doi.org/10.1534/genetics.166.1.351.

Prober, Suzanne M, Margaret Byrne, Elizabeth H McLean, et al. 2015. “Climate-Adjusted Provenancing: A Strategy for Climate-Resilient Ecological Restoration.” *Frontiers in Ecology and Evolution* 3: 65–65.

Rossetto, Maurizio, Jason Bragg, Andrzej Kilian, Hannah McPherson, Marlien van der Merwe, and Peter D Wilson. 2019. “Restore and Renew: A Genomics-Era Framework for Species Provenance Delimitation.” *Restoration Ecology* 27 (3): 538–48.

Sgrò, Carla M, Andrew J Lowe, and Ary A Hoffmann. 2011. “Building Evolutionary Resilience for Conserving Biodiversity under Climate Change.” *Evolutionary Applications* 4 (2): 326–37.

St. Clair, John Bradley, Bryce A. Richardson, Nikolas Stevenson-Molnar, et al. 2022. “Seedlot Selection Tool and Climate-Smart Restoration Tool: Web-Based Tools for Sourcing Seed Adapted to Future Climates.” *Ecosphere* 13 (5): e4089. https://doi.org/10.1002/ecs2.4089.

Williams, Kristen J, Lee Belbin, Michael P Austin, Janet L Stein, and Simon Ferrier. 2012. “Which Environmental Variables Should I Use in My Biodiversity Model?” *International Journal of Geographical Information Science* 26 (11): 2009–47.
